# Advancement in Biopolymer Assisted Cancer Theranostics

**DOI:** 10.1021/acsabm.3c00458

**Published:** 2023-09-12

**Authors:** Tanima Bhattacharya, Subham Preetam, Basab Ghosh, Tulika Chakrabarti, Prasun Chakrabarti, Shailesh Kumar Samal, Nanasaheb Thorat

**Affiliations:** † Department of Food and Nutrition, College of Human Ecology, Kyung Hee University, 26 Kyunghee-daero, Dongdaemun-gu, Seoul 02447, Republic of Korea; ‡ Nondestructive Bio-Sensing Laboratory, Dept. of Biosystems Machinery Engineering, College of Agriculture and Life Science, Chungnam National University, Daejeon 34134, Republic of Korea; § Centre for Biotechnology, Siksha O Anusandhan (Deemed to be University), Bhubaneswar 751024, Odisha, India; ∥ Daegu Gyeongbuk Institute of Science & Technology (DGIST), Daegu 42988, Republic of Korea; ⊥ KIIT School of Biotechnology, Kalinga Institute of Industrial Technology (KIIT-DU), Bhubaneswar 751024, Odisha, India; # Department of Chemistry, Sir Padampat Singhania University, Bhatewar, Udaipur 313601, Rajasthan, India; ∇ ITM SLS Baroda University, Vadodara 391510, Gujarat, India; % Section of Immunology and Chronic Disease, Institute of Environmental Medicine, Karolinska Institutet, Stockholm 171 77, Sweden; $ Nuffield Department of Women’s & Reproductive Health, Medical Science Division, John Radcliffe Hospital University of Oxford, Oxford OX3 9DU, United Kingdom; ○ Department of Physics, Bernal Institute and Limerick Digital Cancer Research Centre (LDCRC), University of Limerick, Castletroy, Limerick V94T9PX, Ireland

**Keywords:** Theranostic, Nanomedicines, Antitumor, Drug Delivery, Nanotherapeutics

## Abstract

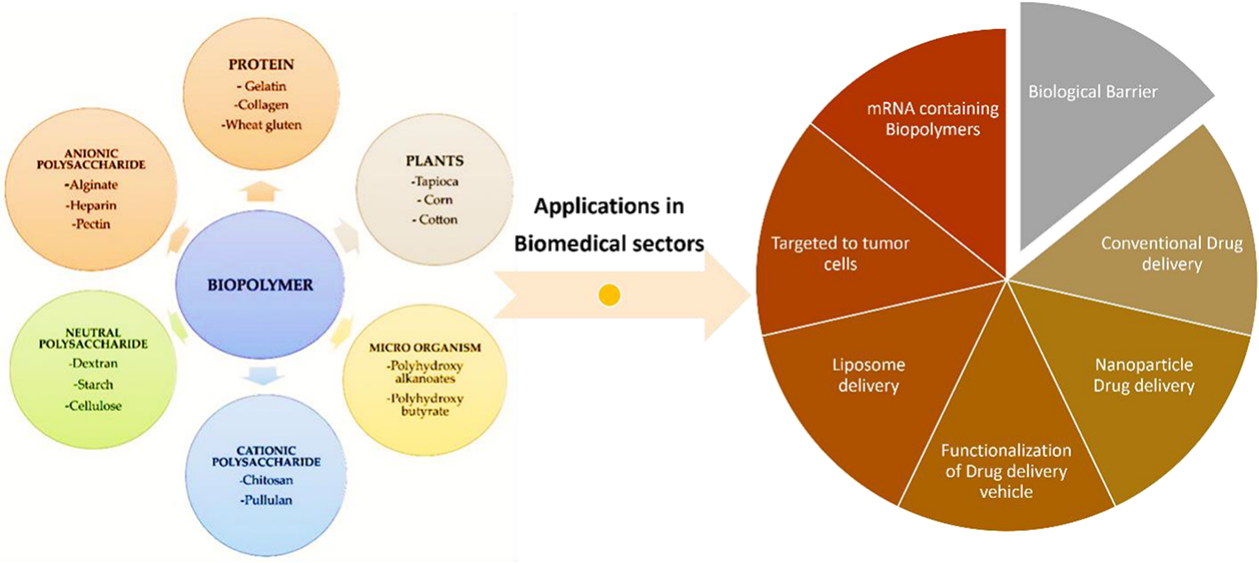

Applications of nanotechnology have increased the importance of research and nanocarriers, which have revolutionized the method of drug delivery to treat several diseases, including cancer, in the past few years. Cancer, one of the world’s fatal diseases, has drawn scientists’ attention for its multidrug resistance to various chemotherapeutic drugs. To minimize the side effects of chemotherapeutic agents on healthy cells and to develop technological advancement in drug delivery systems, scientists have developed an alternative approach to delivering chemotherapeutic drugs at the targeted site by integrating it inside the nanocarriers like synthetic polymers, nanotubes, micelles, dendrimers, magnetic nanoparticles, quantum dots (QDs), lipid nanoparticles, nano-biopolymeric substances, etc., which has shown promising results in both preclinical and clinical trials of cancer management. Besides that, nanocarriers, especially biopolymeric nanoparticles, have received much attention from researchers due to their cost-effectiveness, biodegradability, treatment efficacy, and ability to target drug delivery by crossing the blood–brain barrier. This review emphasizes the fabrication processes, the therapeutic and theragnostic applications, and the importance of different biopolymeric nanocarriers in targeting cancer both *in vitro* and *in vivo*, which conclude with the challenges and opportunities of future exploration using biopolymeric nanocarriers in onco-therapy with improved availability and reduced toxicity.

## 1 Introduction

Cancer is one of the leading causes of mortality worldwide, accounting for nearly 10 million deaths until 2020. Furthermore, World Health Organization (WHO) has predicted that it will rise by up to three times by 2040.^1−3^ Due to lifestyle changes, around 70% of these fatalities occur in low- and middle-income nations. Standard treatment methods for cancer include chemotherapy, surgery, radiation, immunotherapy, and hormone therapy. However, these treatments adversely affect patients.^4−6^ For example, chemotherapy has several hazardous side effects, such as myelotoxicity, leukopenia, cardiotoxicity, blood vessel constriction, and lack of targeted delivery. To overcome these limitations, researchers are trying to find out the potent anticancer drugs with the fewest side effects to develop a better alternative with lower toxicity.^7−11^

Nanomedicine has been fashioned as a dimension of significance in pharmaceutical research, particularly in drug delivery systems.^12−15^ Nanoencapsulation of bioactive compounds is a salient technique for ensuring sustainable bioavailability due to an increase in surface-to-volume ratio through a significant reduction in particle size to a nano level. Biopolymers with dimensions ranging between 10 and 1000 nm can be used as nanoparticles (NPs).^16−20^ Recently, many researchers have sought the attention of biopolymers due to their biocompatibility, easy design and preparation, surface variations, and interesting biomimetic characteristics.^21^ A few recent studies through the application of nanomedicines from natural sources encompassing lipid nanocarriers for phenolic drug delivery,^19,22−25^ polysaccharide nanocarriers^26^ and composite nanocarriers for better specificity, prolonged circulation, better bioavailability, and enhanced permeation rate (EPR) have been discussed. These polymers have created a unique niche and inefficient ability in drug delivery. However, the possibility of synthetic polymers exhibiting cytotoxicity should not be overlooked. Gopi et al. performed a detailed study on the mechanism and role of effective drug delivery systems.^27,28^

**Figure 1:**
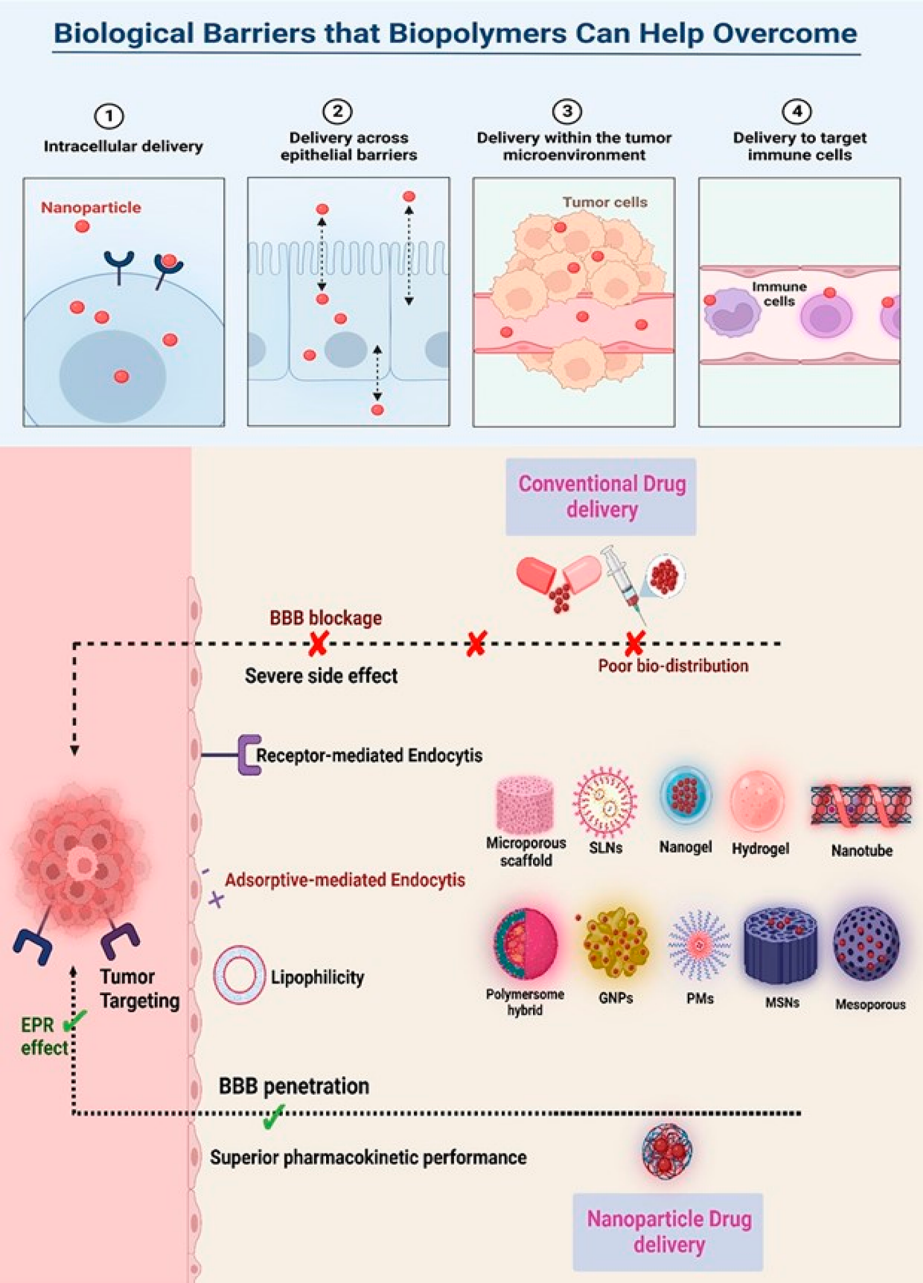
Schematic representation of biopolymers used to overcome the traditional barrier methods from conventional drug delivery methods. EPR enhances tumor-killing activity efficiently.

Moreover, the application of polymer conjugates for nanomedicines has been reported in the past decades. The application of nanosystems facilitates the delivery of drug agents in a systematic manner at the targeted site. Calzoni et al. mentioned various kinds of biopolymers used to formulate nanomedicines for controlled release formulations for anticancer therapy, as shown in Figure 1.^29−33^ Many researchers have also tried to develop a series of polylactic acid (PLA) conjugates through functionalization click chemistry for attaining better results in target binding and enhanced penetration of drugs to the target site.^34−37^ Recently, therapeutic and diagnostic methods based on a biopolymer have demonstrated great promise for improving cancer treatment. Significant advancements in cancer detection, prevention, and therapy focus on a new field of integrated study in biology, chemistry, engineering, and medicine known as cancer nanotechnology.^38−41^ Due of their increased efficacy and safety, biopolymers have drawn the attention of scientists in recent years. Recent developments in nanotechnology assisted anticancer medications include Myocet (Perrigo, Dublin, Ireland), DaunoXome (Gilead Sciences, Foster City, CA, USA), Doxil (Johnson & Johnson, New Brunswick, NJ, USA), and Abraxane which have been authorized by the US FDA as a result of these applications (Celgene, Summit, NJ, USA).^42−49^

This review focuses on various techniques of nanodrug design, mechanisms, different biomaterials used, photodynamic therapy, and image analysis in an *in vivo* model. In addition, this review also explores the possibility of using novel biomaterials for antitumor/anticancer agents and PDT in *in vivo* models.

## 2 Characterization and Synthesis of Biopolymeric NPs Used in Drug Delivery Systems (DDSs)

**Figure 2:**
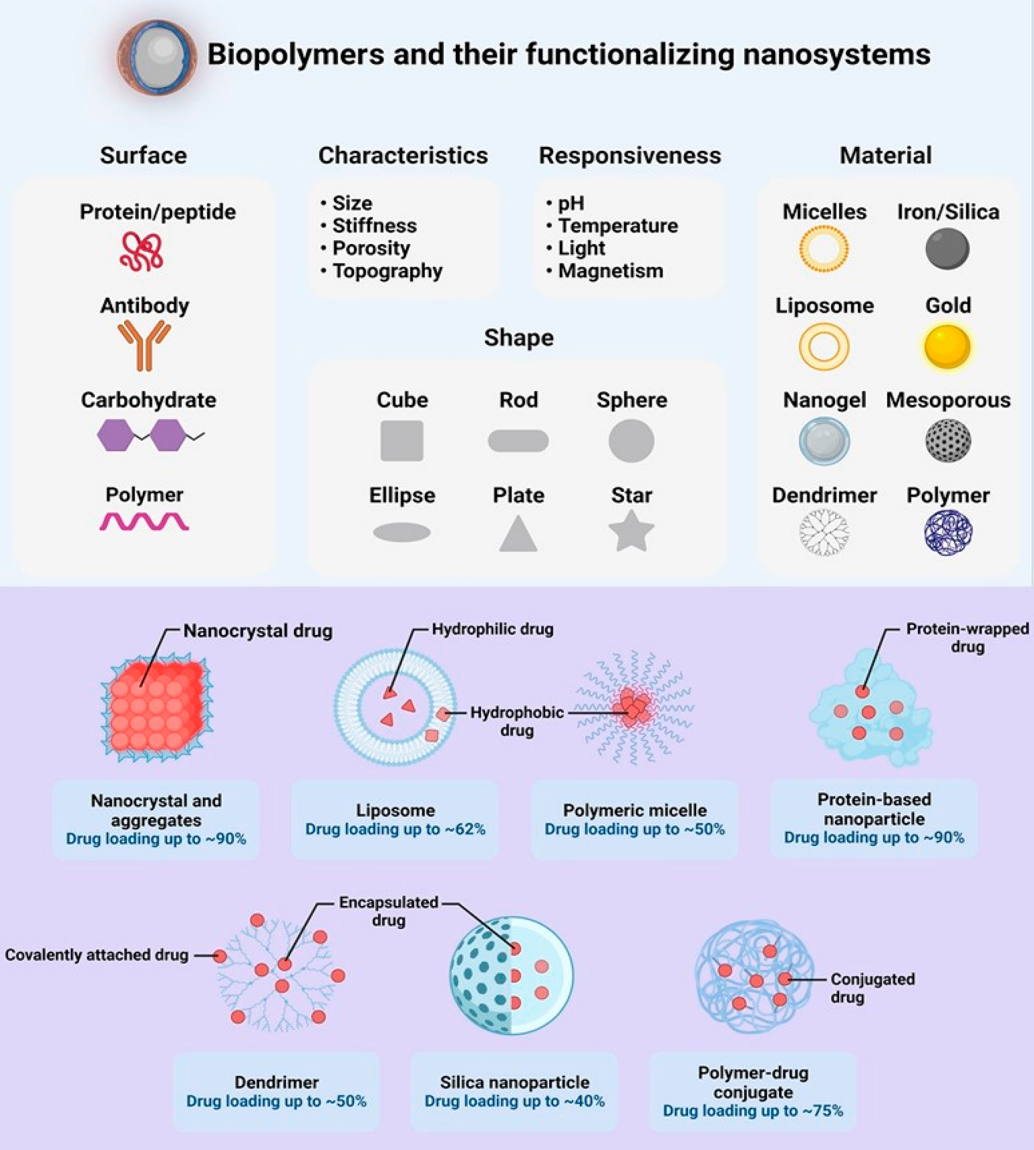
Graphical representation of different kinds of biopolymers and their working nanosystems.

Nanocarriers (NCs) loaded with anticancer drugs have several advantages over free drugs. They protect drugs’ premature degradation and nonspecific interactions, thereby ensuring target-specific killing of tumor cells.^50−52^ The biocompatible feature of NCs is of prime importance, and this feature makes the NCs more effective in enhancing efficacy and extending the shelf life of the drug in circulation.^53^ In this review, our focus is on the biopolymers that are currently being used as NC-DDSs. Biopolymers are naturally occurring substances possessing nontoxicity, biocompatibility, and biodegradability properties. To date, only polymers have been researched for NC formulation development.^54−57^ Polymers can either be synthetic or natural, wherein natural polymers such as chitosan, silk, alginate, albumin, starch, carbohydrate, proteins, and lipid materials may be incorporated for encapsulation without the requirement for chemical modification of the drug.^48,58^ Several formulations and techniques of the biopolymeric NPs resulted in efficient nanotransporting properties, which exhibited excellent antitumor effects in the past few decades. From Table 2, we can obtain insight into the techniques that depict the effect on particle size for biomedical applications, shown in Figure 2.^47,59−61^

**Table 1:** Surface Modifications of Nanoparticles by Biopolymers for Enhanced Drug Delivery

**Nanoparticle**	**Biopolymers**	**Drug**	**Biomedical Applications**	**References**
PEG	Lipid	Paclitaxel	*In vitro* uptake enhanced	(80)
Hydroxylated ethyl starch	PEG layer and mannose	N/A	Targeting dendritic cells	(81)
MMT clay	Starch/d-l-lactic acid	DMSO (dimethyl sulfoxide)	*In vitro* studies revealed sustained release	(88)
Gold nanoparticles	Gelatin	Doxorubicin	*In vitro* studies revealed sustained release	(89)
Gold nanorods	Lipids	N/A	Enhanced bioimaging	(90)

**Table 2:** Nanoparticle Formulation Techniques to Understand the Processes That Provide the Desired Particle Size for Biomedical Applications

**Techniques**	**Nanoparticles**	**Dimension**	**References**
Electrospinning	Elastin-like polypeptides	110–680 nm	(109)
Polymerization under nonlinear substructuring	Gelatin core with quantum dot on surface	30–100 nm	(107)
Low energy throughput (mechanical stirring @800 rpm without heat)	PEGylated NP	174–184 nm	(108)
Enzymatic synthesis	Polyglycerol adipate	136 nm	(70)

### Lipid-Based Biopolymer

**Figure 3:**
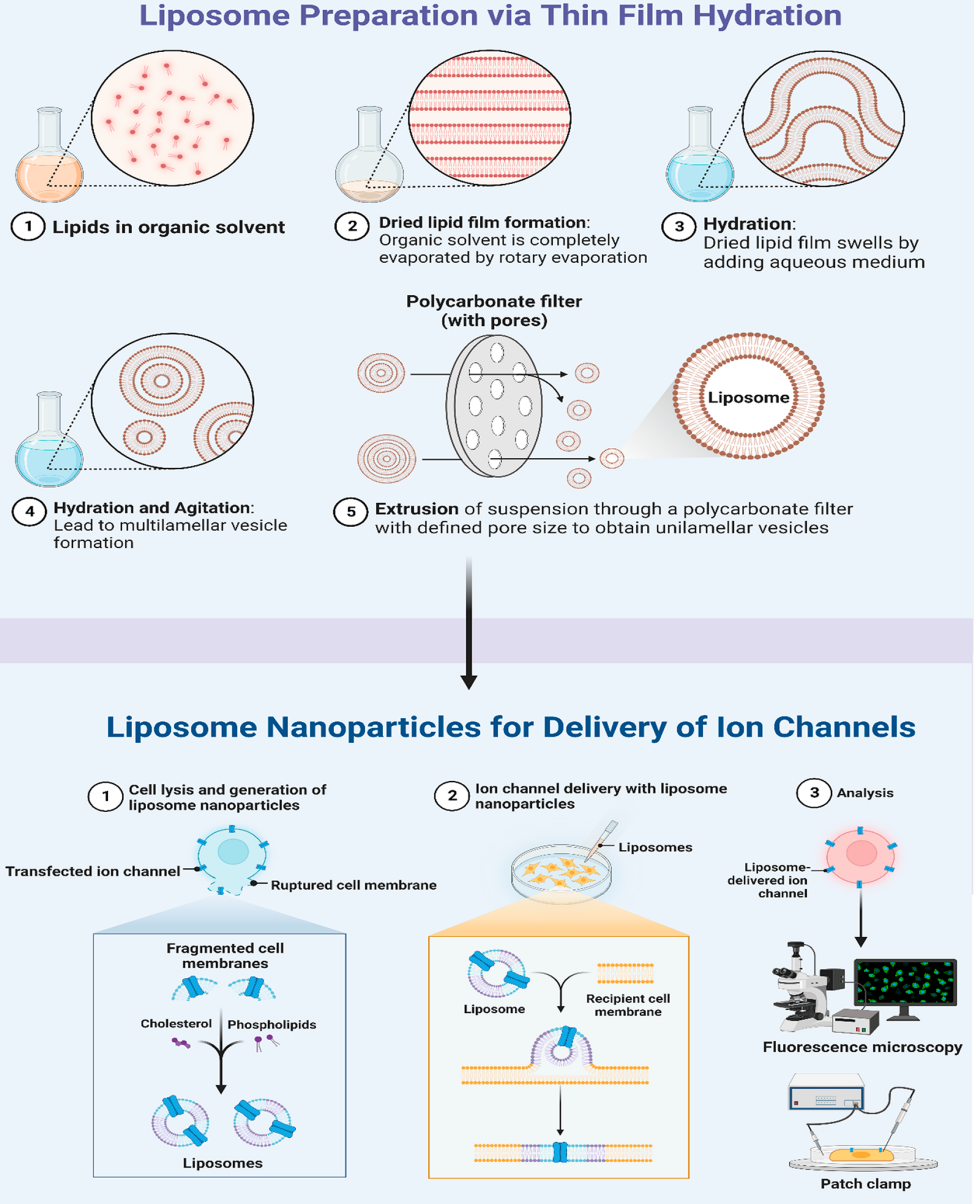
Liposome preparation and nanocarrier production using film hydration and ion channel methods.

The use of organic polymers has already been practiced in the last few decades.^62^ Organic polymers, including solid lipid NPs, liposomes, and proteins, have been considered suitable nanocarriers in DDSs.^57,63,64^ Lipid polymers are commonly applied, possibly due to their efficient capacity to encapsulate both hydrophobic and hydrophilic drugs.^65−68^ Liposomes are self-assembled concentric lipid bilayers with an aqueous core. Recently, lipid nanoparticles (LPNs) have been used to deliver water-insoluble drugs.^69^ Weiss et al.^70^ showed that lipid material such as stearic acid-modified polyglycerol adipate (PGAS) can be used as a promising carrier for drug delivery and did not require surfactant.^70^ Only coating covalently or non-covalently with *N*-(2-hydroxypropyl) methacrylamide (HPMA) copolymers was performed.^70^ These nanoparticles have equivalent particle sizes but sometimes exhibit lower or minus zeta-potentials.^70^ Double labeling of the NPs was done with the fluorescent dyes DiR in a non-covalent manner and DYOMICS-676 which was bound covalently to a copolymer of HPMA. The bio distribution was investigated noninvasively through multiple-spectra-based optical imaging. Both coatings caused variations in the pharmacokinetics and bio distribution of healthy and cancer-bearing mice. We have already discussed the spray drying technique in the formulation of NPs; nowadays this technique is being modified by electro-hydrodynamic atomization,^71^ as shown in Figure 3.

Zhen et al. observed a successful gene delivery using crystalline lipids coupled with a photosensitive agent, and this combination has been proven to be an excellent nanotheranostic agent.^72^ Although lipid NPs have created a domain in nano-DDSs, their delivery and release of drugs at tumor sites are found to be erratic. Several techniques were thus applied to enhance drug delivery, the significant one being the ultrasound mechanism adopted by Nahire et al.^73^ They observed a 76% targeted drug release when the lipid nanocarrier is in the presence of cytosolic glutathione.^73^ However, if the nanocarrier is subjected to 3 MHz for 2 min, then the release of the drug was found to increase to 96%. Thus, from the above scheme presented by Nahire et al., it is evident that lipid NPs can be used as an effective DDS and in ultrasound imaging through modification of their environment.^73^ The current trends to enhance the efficacy of anticancer drugs are remarkable. Despite having favorable characteristics of lipid nanocarriers in therapeutic delivery, they also have several drawbacks such as the absence of quality control and self-assembly formulation inconsistency leading to inhibition of therapeutic efficacy at the target site.^74^ Thus, to overcome such issues, researchers reported new dimension of LNC synthesis using the electrospray ionization mass spectrometry (EIMS) fragmentation along with matrix assisted laser desorption/ionization (MALDI) and time-of-flight (ToF)-based mass spectrometry.^75^ A reliable synthesis of pure phospholipid peptide bioconjugates (peptide amphiphiles) was developed.^76^ This technique avoided unwarranted hydrolyzed byproduct formation that could dampen the therapeutic efficacy of the polymorphic nanotherapeutics.^56,77,78^

**Figure 4:**
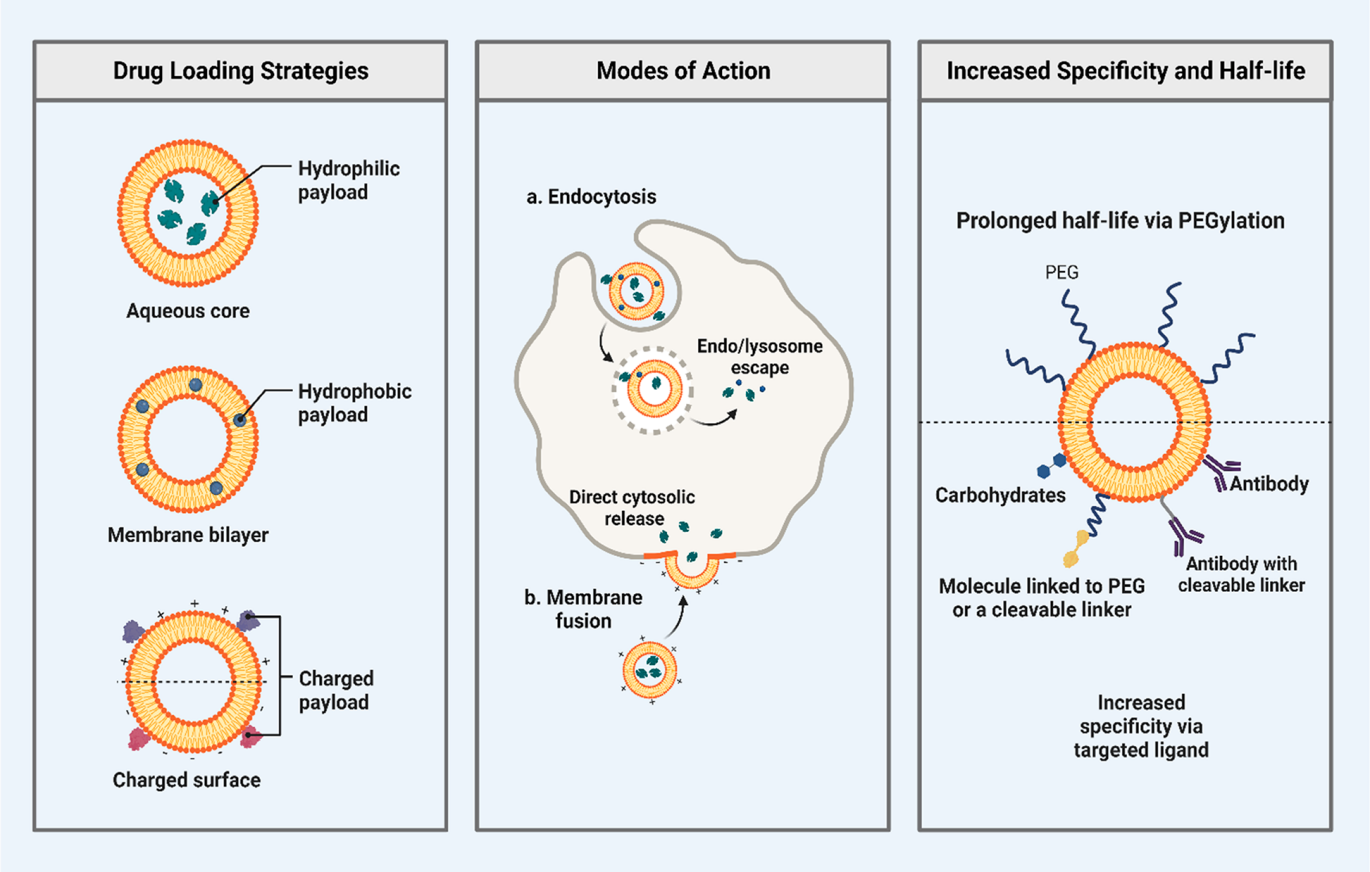
Drug loading activity and mode of action in a cell, with a proper strategic mode of action and representing specificity to target cells.

Recent biopolymer approaches in DDSs are being introduced wherein the nanocarriers cause a half burst release before accumulating at the tumor site, thereby causing toxicity due to the circulation of materials.^79^ Therefore, researchers have shifted focus to stimuli-responsive carriers. Michelle Stollzoff et al. reported a novel, lipid-coated nanoparticle which is pH sensitive and provides a 100–1000 nm expansion in size when placed in a mild acidic pH environment and in the presence of PEGylated lipids (PEG = polyethylene glycol).^80^ The modification of the surface PEG-L-eNPs allows the introduction of folic acid (FA) and folate receptor targeting.^80^ Therefore, these resultant polymer/lipid hybrid nanocarriers, FA-PEG-L-eNPs, provide an enhanced potency and uptake when loaded with paclitaxel *in vitro* and when compared to nontargeted PEG-L-eNPs.^80^ Another study on surface modification of the nanocarrier for better targeting was also reported by Biao Kang et al.^81^ These researchers utilized hydroxyethyl starch to prepare the PEGylated nanocarrier and modified the outer PEG layer with mannose to target dendritic cells, as shown in Figure 4.^81^ The human plasma interaction and a distinct pattern of protein adsorption and enhanced affinity ensure better targeting behavior with dendritic cells.^81^

Researchers are now developing self-organizable assemblies along with varying amphiphile phase structures with polymer NPs, as this is believed to be promising for designing and developing efficient nanocarriers.^82^ Angayarkanny et al. developed assemblies of micelles containing lauryl esters of tyrosine (LET) coated with polymer nanoparticles which behave as potential nanocarriers for the model solid lipid stearyl alcohol.^83−86^ It is essential to mention that amino acid surfactant dispersions in pure lauryl ester of tyrosine and lauryl esters of phenylalanine micelles separated spontaneously, thus suggesting a minuscule encapsulation of amino acid surfactant in the micelles.^83−86^ Through this, sufficient proof has been obtained to believe that polymer-coated LET micelles act as suitable matrixes for SA encapsulation, the strength of which is attributed to a H bonding interaction between the phenolic group present in LET and the hydroxyl (OH) group current in the SA.^83−86^ Many researchers are thus keen to develop NPs for anticancer therapy from natural silk.^87^ The silk NP was depicted by F. P. Seib et al.,^87^ who conducted an *in vitro* study of silk NPs loaded with doxorubicin to establish the nontoxic property of NPs on healthy cells with increased efficacy that overcomes the drug resistance mechanism, which is shown in Table 1.^87^

### Polysaccharide-Based Biopolymer

Polysaccharides have been found to be significant components for stimuli responsiveness in DDSs based on them being biocompatible and less toxic.^91^ Alginates and chitosan (polysaccharides) are safe for use alone or with specific surface modification in DDSs.^92−94^ Wang et al.^95^ suggested some interesting manufacturing and surface modification techniques to formulate the biopolymers like polylactic acid and chitosan NPs for nanomedicine application.^95^ The processes for PLA involved the emulsion diffusion method where the fabrication of both single emulsion and double emulsion systems was undertaken. This technique was undertaken for the formulation and development of lipophilic (hydrophobic) substances, while the other method adopts (i) hydrophilic chemical entrapment: the salting out method, where aqueous and organic phases are emulsified in O/W and further distilled water was added which resulted in raw NPs, (ii) nanoprecipitation: where a syringe pump is used in the oil bath containing the magnetic stirrer and (iii) the emulsion evaporation method: where drug agent is dissolved in the polymer in organic solvent in which the emulsion is carried out in high shear phase and further followed by solvent evaporation in a vacuum resulting in NPs. The chitosan NPs (CNPs) were developed using the ionic gelation method whereby sodium tripolyphosphate is added to the chitosan solution and homogenized at high speed forming nanoparticles. For the reverse micelles method, chitosan with cross-linking agent is added to surfactant dissolved in organic solvent, stirred overnight, and later purified to obtain CNPs. These CNPs were also obtained through applying the compressed air to the chitosan solution, known as the spray drying technique. In addition to that, a hot air chamber and blow-drying chitosan into an alkali solution is known as the coacervation process at an adjusted pH of more than 6.5. Surface modification techniques of the nanopolymers by improving hydrophilicity, chitosan functionalization, target functionalization, pH-sensitive coating, and plasma treatment under pressure gauge in a gaseous environment presented a better result in *in vivo* treatment. These were thereby followed by observational studies of addition of trimethyl chloride, galactoside, polyethene glycol, thiolation, and target modifying agents to chitosan.

Alvarez-Lorenzo et al.^96^ showed that ionic polysaccharides exhibit sensitivity to pH and this ion sensitivity is transferable physically or chemically through cross-linking networks. Chitosan, the only naturally available cationic polysaccharide, tends to swell at acidic pH and shrinks at neutral/alkaline pH, thus assisting in faster drug release at acidic pH. The reversal of this property is observed with anionic polysaccharides. The osmotic effect of ions enhances the cross-linking density, thereby enhancing the release rate. Owing to ionic interactions, an affinity-controlled mechanism takes place, which results in the release of the drug. These features thus make polysaccharides a suitable choice for site-specific oral drug delivery, particularly for release into the colon. Therefore, resistance to the degradation of the drug by the enzymes of the upper gastrointestinal tract and susceptibility to enzymes present in the large intestine may thus synergistically cause efficient site-specific release. The report also mentioned that the variations in pH between healthy cells and tumor cells can also lead to the triggering effect of the carriers. The electric field may also be essential in drug release’s operating cycles. The other factors that can act as a stimulus, such as light, temperature, and redox reactions, can also affect release rate. Polysaccharide-based NPs have significant usage in theragnostics. Surface modifications through self-assembly and cross-linkers are seeking more attention nowadays. Researchers have been trying to industrialize polymeric nanocarriers. Kim et al.^97^ synthesized and developed an uncoated, chitosan-based coating and starch-based coating of magnetic NPs to use as a hyperthermic thermo seed. The chitosan-coated magnetic NPs were synthesized at 23 °C in the presence of an alternate current (AC) magnetic field compared to the starch-coated magnetite. The particle capture rate was 96% when an external magnetic field of 0.4 T was present. As the normal viability of 93.7% of L929 cells was compared to that of the KB carcinoma cells, the rate of capture of the KB cells relatively surged by 10.8% with the chitosan-coated magnetic NPs. The *in vitro* studies revealed that chitosan-coated magnetic NPs showed high compatibility, thus proving beneficial and significantly promising for use in magnetically targeted hyperthermia.

The latest work by Alkanawati et al.^98^ depicts a method of laboratory scale production of nanocarriers which can indeed be applied to an industrial scale for efficiency of production of such nanocarriers. They successfully produced nanocapsules of enhanced quality (low PDI and batch variability) along with a 300-fold surge in product output. However, the size of the produced nanocarriers could be easily manipulated through variations in the operating conditions. These findings were coupled with the knowledge that inline implementation of microfluidization could provide a beneficial way for the upscale production of nanocapsules for delivery of the drug at an industrial level.^99^ Suarasan et al.^89^ developed a doxorubicin-loaded nanocarrier made up of gelatin and Au NPs. The free drug showed acute toxicity and self-chemical modifications. Thus, loading the encapsulated drug particle between the positively charged gelatin layer and the negatively charged Au NPs avoids the requirement of any chemical change of the drug, which further ensures maximal antitumor activity. Furthermore, gold nanorods are being implanted in lipid nanodiscs, which can be enhanced by clustering and be used in sensing and imaging applications.^90^ Acetylated rapeseed protein isolate (ARPI) with chitosan coupled anticancer drug doxorubicin (DOX) are being designed as nanocarriers along with cathepsin B. Cathepsin B is a lysosomal acid protease that is ubiquitously expressed in all lysosomes of mammals and is enormously upregulated and active in various kinds of malignant cancers. The bioactive peptides that are ARPI-derived exhibit an anticancer effect synergistically with DOX through regulation of various pro- and anti-apoptotic proteins such as *p53*, *Bax*, *Bcl-2*, and *pro-caspase-3* which are present in the mitochondria. In a CathB-overexpressing orthotropic breast tumor model, DOX-ARPI/CS NPs significantly inhibited tumor enlargement, enhanced tumor cell apoptosis, and increased hosts’ survival without producing any systemic toxicity.^90^

Polysaccharides, including chitosan and contrast agents, are also actively used in drug delivery and in image guided radiation therapy.^100^ Starch being biocompatible, inexpensive, readily available, and biodegradable has sought attention in DDSs as an alternative nanocarrier. Its hydrophilicity, however, can sometimes create problems in stability, and thus, acetylation may be required. To bypass this, Namazi et al.^88^ suggested using montmorillonite (MMt) clay, a biodegradable nanomaterial, along with d and l lactic acids as a better substitute. Reports showed high thermal stability of the MMt-induced nanocarrier and an efficient encapsulation and drug release pattern. A similar statement was also observed by a mini-review of cellulosic starch being reinforced by nanocrystals to enhance the biocompatibility of the polymers.^101^ Mukwaya et al.^102^ discussed saccharide-based nanocarriers for DDSs. According to their work, saccharides exhibited good encapsulating efficiency and low toxicity to increase the drug accumulation in the targeted site for a prolonged period.^102^ One- or two-pot syntheses have recently been developed for polysaccharide nanocarrier synthesis.^102^ This is inclusive of the self-assembly of nanovectors through reversible fragmentation chain transfer addition or anionic polymerization.^102^ However, these techniques possess certain limitations due to their nonemployment in the biomedical nanocarrier synthesis originating from natural macromolecules, which makes free radical copolymerization unique compared to other techniques due to its ability to be utilized in purely aqueous media devoid of surfactants.^102^

Nanomaterials are much more efficient than chemotherapeutics in delivery to the target site.^103^ However, further research is being conducted to improve the penetration of large sized NPs through introducing cross-linking methods.^104−106^ Ju et al.^107^ reported the formulation of a nanogel containing a polyelectrolyte core cross-linkage comprised of *N*-lysinal-*N*′-succinyl chitosan and poly(*N*-isopropylacrylamide) along with a cross-linked shell of bovine serum albumin which showed swelling in an acidic environment followed by shrinking under neutral conditions.^107^ This swelling was responsible for quickly releasing the encapsulated chemotherapeutic agent into the cancer cells for effective cytotoxicity. These nanogels also had a better effect on the neighboring cells in the vicinity of the tumor site. The design and development of a unique nanomedicine by Ren et al.^108^ showcased several advantages such as ease of preparation and reproducibility, increased payload of drug, superior and enhanced stability, prolonged circulation, and enhanced therapeutic effect.^108^ The high reproducibility rate of the 18 assemblies of molecules should, therefore, motivate the design and development of new nanomedicine, which was done through nanoparticles that are self-assembled devoid of aid of surface-active substances and based on the conjugation of docetaxel to d-α-tocopherol succinate.^108^ Reduction-sensitive prodrugs were developed through synthesis with introducing a disulfide bond into the linker and were compared with reduction-insensitive prodrugs as the control.^108^ The investigation of the morphology and stability of self-assembled nanoparticles was done. The researchers were keen on developing nanomedicine from biodegradable polymers due to their enhanced compatibility and bioavailability.^108^ They were also approved by the FDA and the European Medicine Agency (EMA), thus making them great candidates for clinical oral and parental administration. Wu et al.^109^ reported using formulations of NPs comprised of elastin-like polypeptides (ELPs) that are genetically engineered. They possess characteristics of biodegradability, biocompatibility and bio responsiveness.^109^ The study involved using NPs ranging from 300 to 400 nm in diameter and ELPs and drugs which are priorly co-dissolved in an organic solvent followed by acceleration through a gradient-based voltage.^109^ They are then dried through the process of evaporation during transit and collected from the surface of the target.^109^ The results indicate that the diameter and size of the particle, polydispersity, and morphology strongly correlate to the solvent concentration, voltage of spraying, and polymer’s molecular weight.^109^ Surprisingly, drug loading at 20 w/w% did not affect the morphology of the particle. An aspect that is noteworthy with this technique is the fact that the release of drug from these particles was related to pH-based solubility of the ELPs of the parent which suggests that electro spraying is a flexible and efficient technique for the generation of stimuli-responsive drug NPs.^109^

Free radical copolymerization’s eco-friendly, inexpensive, and straightforward characteristics allowed for developing a one-pot synthesis scheme for nanocarriers named “graft copolymerization-induced self-assembly” (GISA).^110^ Using a two-pot approach involves a modification in methodology and the usage of postmodification or postpolymerization self-assembly-based techniques.^111^ Techniques such as micellization, nanoprecipitation, dialysis, solvent evaporation, and emulsification can be used for the synthesis of nanocarriers from polysaccharides.^112^ However, the one-pot approach is preferentially utilized for the doxorubicin drug delivery at the anticancer site. The development of biodegradable drug carrier formulation by the electrospinning method was reported recently.^113^ Electrospinning is another technique in which the particles are synthesized by applying an electrohydrodynamic force that is uniform to break up liquids into fine jets. This is an exciting technique for quick and increased production of NPs that possess a controlled morphology that can assist with controlled release in the drug delivery of cancer. This resultant product of NPs produced through electrospinning must display (1) reliable entrapment of the drug, (2) uniform particulate size, (3) sufficient stability under physiological conditions, (4) adequate release or in response to a stimulus in a time-dependent manner, and (5) biological compatibility.^114,115^

## 3 Mechanism of Drug Delivery through Biopolymer

**Figure 5:**
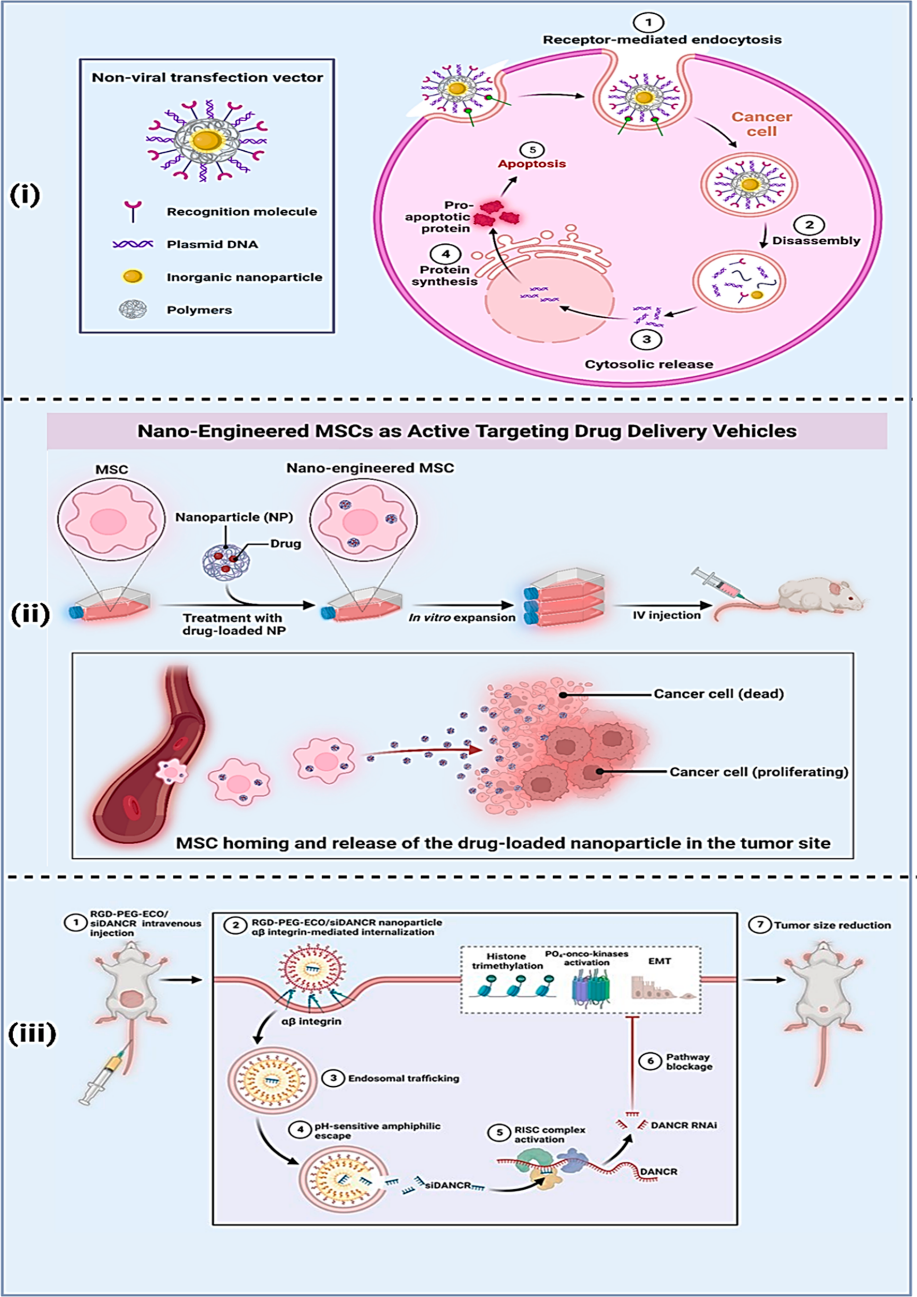
Biopolymer assisted nanodrug delivery mechanisms: (i) nonviral transfection methods, (ii) nanoengineered MSC methods, and (iii) integrin mediated initialization to the targeted tumor cells.

Many studies were reported which were based on the usage of polymeric materials for drug delivery.^116−119^ Biopolymers can be injected in the body via syringe, and once injected into the body, they solidify and form a depot of semisolid nature.^120^ Depending upon the solidification process, injectable implant systems are divided into five types: thermoplastic pastes, cross-linked systems *in situ*, gelling systems that are thermally induced, precipitates *in situ*, and hydrophobic lipophilic fatty acid-based injectable pastes.^120^ The method for studying this involves the process of extraction of solvent around the surrounding tissue resulting in precipitation which protects the protein and amide-based polymers from being damaged by heat and shear stress (shown in Figure 5).^120^

Biocompatibility and biodegradability are desirable properties of the materials used in the development of nanocarriers.^121^ Various natural polymers have been investigated, like starch, cellulose, chitosan, albumin, and gliadin fulfilling these requirements.^122^ Starch has already been transformed into NPs and is used widely in delivery of drugs.^123^ For better efficacy, instead of direct targeting, doxorubicin can be delivered into the DU145 cell by entrapping into the hydroxyethyl starch, which is a synthetic polymer derived from starch.^124^ It enables the drug to circulate through the blood in a prolonged period.

M. Gulfam et al.^125^ extracted gliadin particles from gluten by the desolation method, which were already being used for drug delivery earlier. However, they resulted in low drug loading efficiency and less ability to separate the particles from the aqueous phase.^125^ Therefore, researchers processed the gliadin NPs through the electrospinning technique, which does not require the utilization of a surfactant. Gliadin NPs and gliadin gelatin nanocomposites loaded with cyclophosphamide were the target NPs in the drug delivery for this study.^125^ The study revealed that cyclophosphamide was slowly released from nanoparticles of gliadin for 48 h, and composite nanoparticles of gliadin–gelatin caused drug release rapidly. Moreover, 24-h cultured breast cancer cells with 7% gliadin nanoparticles loaded with cyclophosphamide became apoptotic.^125^ Also, the downregulation of the Bcl-2 protein was confirmed through analysis by Western blotting. As a result, this anticancer drug-loaded gliadin nanoparticle can be used as a powerful tool for cancer therapeutics.^125^

A DDS utilizing polymers extracted from the exoskeleton of crustaceans or any shell animals is also the most versatile substances chitosan.^126^ According to Uchegbu et al.,^127^ research of chitosan drug delivery began in 1990. Chitosan is deacetylated chitin (the exoskeleton of many animals). It has shown promising results as a film forming material required for drug delivery.^127^ The amphiphilic character of chitosan provides the ability to form NPs in aqueous solution instead of using any cross-linking agents or ionic gelation agents.^127^ The CNP less than 1 μm in dimension is stable for six months after drug loading.^127^ Chitosan NPs showed oral bioavailability of hydrophobic drugs up to 6-fold and when bound to anticancer agents effectively delivered the drug to the tumor site by exhibiting tumoricidal activity without being toxic to normal cells.^127^ When 200 nm dimension NPs were also administered to rabbit retinal cells, they were nontoxic, showing benefit as an ocular agent. It is also able to deliver peptides to the brain through the intravenous and oral route.^127^

Another new investigation was performed by Yi Tian et al.^128^ using silk fibroin. They developed silk fibroin NPs through a magnetizing method from the cocoon fiber of *Bombyx mori*.^128^ The past decade has witnessed the application of silk fibroin NPs as a DDS for release and entrapment of drugs. It has also shown its efficacy as a lysosomotropic anticancer nanodrug carrier when loaded with doxorubicin and has overcome multidrug resistance.^128^ However, the nonspecificity of tumor targeting could bring about an accumulation of therapeutic agents. Therefore, a single-step potassium phosphate salting-out technique included certain amounts of magnetic iron oxide NPs (MNPs) in phosphate solution that were hydrophilic in nature.^128^ As a result, the magnetic silk fibroin NPs loaded with doxorubicin showed suppressed tumor growth and a 100% survival rate even on day 30. Therefore, testing this in an *in vivo* model can serve better for clinical trials in the future.^128^ Mohapatra et al.^129^ studied a new formulation incorporating magnetic NPs in chitosan along with the antibiotic vancomycin to observe drug release through magnetic stimulation. The MNP embedded in chitosan showed more effective drug release in a controlled manner as compared to the nonstimulated one.^129^ These characteristic features would greatly benefit clinicians to have control with drug delivery, dosage timings, and local concentration which could be tailored to the clinical needs of the patient.^129^

**Figure 6:**
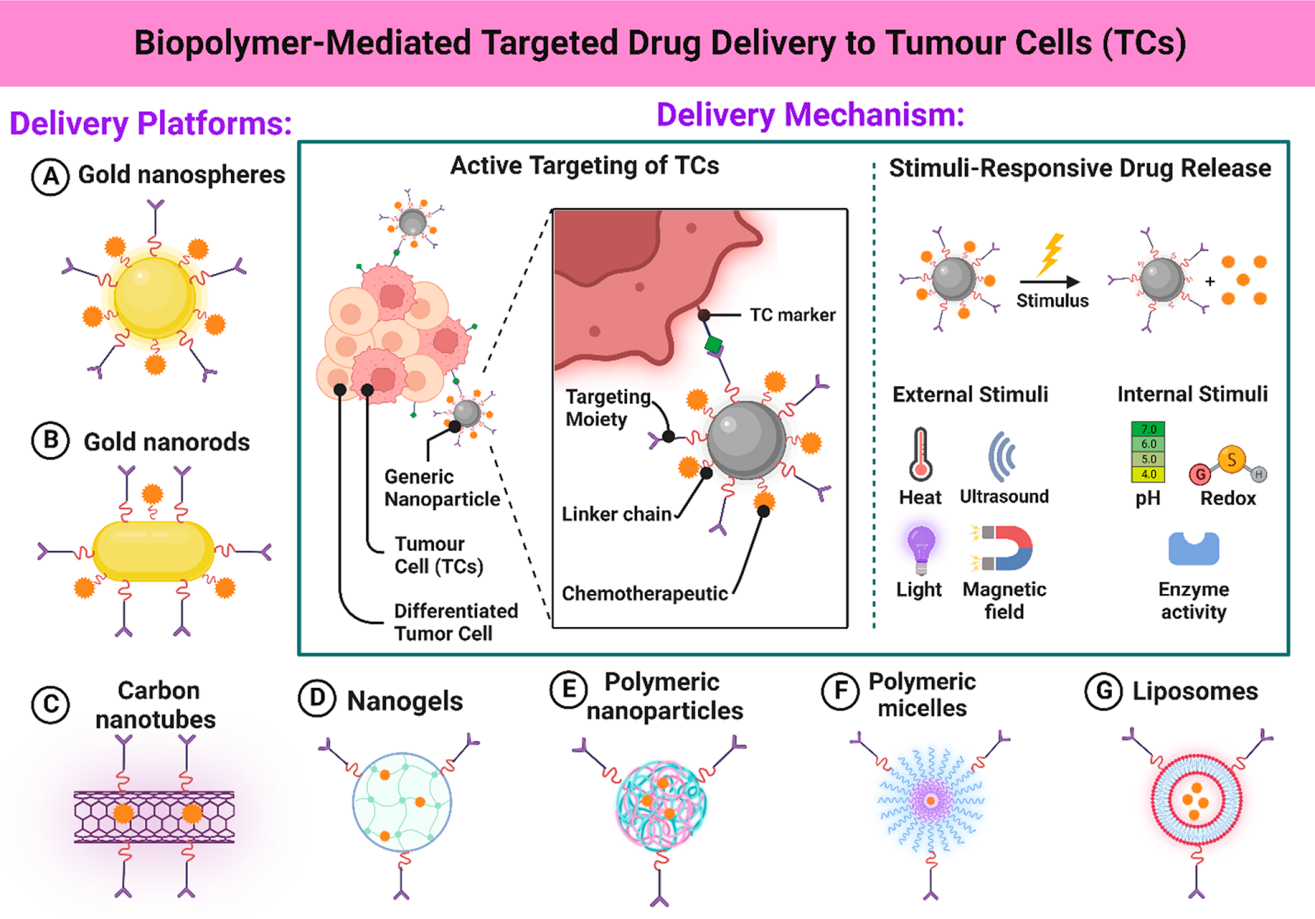
Biopolymer-based targeted drug delivery inside the tumor cells, explanting the mechanism via different stimuli responsive platforms.

Sun et al.^130^ critically reviewed the rational design and analyzed nanocarrier properties in DDSs. After intravenous administration, he concluded that nanomedicine functions through a five-step CAPIR mechanism. This includes (1) circulation through the compartments of blood, (2) accumulation and aggregation in the tumor tissue through its leaky, hastily built vasculature, (3) greater penetration into the tissues of tumors, (4) internalization, and (5) intracellular drug release .^130^ Further, researchers also observed that the nanomedicines involved per step differ in surface charge, dimensions, and stability dilemmas, thereby proposing accumulating these properties into one nanomedicine (3S transitions for short) to eliminate such dilemmas.^130^ With reference to the previous context and through understanding of CAPIR and 3S transitions of nanocarriers several modifications on the surface, stability, and size are being conducted.^130^ Moreover, many researchers have synthesized amphiphilic self-assembled glycyrrhizic acid-biotin-starch NPs (GaBS NPs) through a single-step esterification reaction and they observed the cellular uptake of free doxorubicin as well as GaBS NP loaded doxorubicin.^130^ The cellular internalization of drug loaded NPs was confirmed to be more rapid than that of the pure drug. A surface modification study was reported by Simon et al.;^131^ they synthesized a completely carbohydrate-based nanocarrier through surface-based modification using various sugar derivatives (hydroxyethyl starch, dextran, or glucose) through copper-free click chemistry.^131^ This study revealed a strong interaction between sugar moieties and plasma proteins.^131^ The cellular uptake showed no nonspecific interaction between the carbohydrate NPs and phagocytic cells.^131^ The advancement of peptide-based nanocarriers was also discussed by Wei et al.^132^ According to them, peptides have multiple biomedical applications, like self-assembling them into nanocarriers in drug delivery, or as a targeted ligand for tumor epithelial cells, or for enhancing the penetrating ability of drug loaded in them at the tumor site, or a triggering enzyme-responsive group to create a microenvironment for the tumor to resolve the problem of nanoparticle degradation.^132^ This study also revealed that sometimes there are limitations of these NPs as drug agents due to their inability to accumulate at the tumor site and penetration issues, which peptides or surface modified peptides can resolve,^132^ as shown in Figure 6. The pancreatic cancer cell target by some active biomolecules entrapped in pectin derived peptide by Sanna et al.^133^ formulated some quinoxalinediones and entrapped them into pectin derived peptide to observe their anticancer efficacy. Antiproliferative studies were performed where the targeted NP proved to be more potent than the nontargeted one.^133^

Nanopolymeric materials exhibited a better and more effective drug delivery than chemotherapeutic agents.^134^ However, there were certain limitations about the penetration of nanocarriers into solid tumors of big diameters.^36^ Traditional polymers can only penetrate up to 50 μm; however, nanopolymers associated with anticancer agents can penetrate into tumors of 100 μm diameter.^135^ Dextran nanopolymers embedded in aldehyde functionalized doxorubicin NPs (aldehyde dextran doxorubicin conjugates) showed such properties. 2D nanocarriers’ monolayer studies also provided huge discrepancies when administered into *in vivo* models.^135^ As the efficacy was insignificant, the developed 3D cell structure was used to better understand drug (doxorubicin) release with anticancer therapy for use in childhood stage. The drug use to eradicate the extra cranial tumor (neuroblastoma) in the expensive *in vivo* model, time consumption, and discrepancies.^135^ The role of the glycocalyx in the uptake of the ald-dex-dox NPs was also studied. The study involved understanding the interaction between the dextran-based nanocarrier and the highly glycosylated tumor cell surface and their penetration ability.^135^

Wang et al.^136^ extensively reviewed drug loading strategies for nanocarriers. They stated that major nanocarriers are developed to overcome problems of hydrophobic drugs, and researchers mainly focus on environment or on specific components without paying much attention to drug loading capacities.^136^ There are three types of loading capacities of nanocarriers wherein each system possesses its own release mechanism. In the case of the loading system on the surface, the loading of the drug is dependent upon absorption, while its release depends on desorption.^137^ The matrix loading system is determined by degradation of the carriers or diffusion since molecules of the drug are embedded in the nanocarriers.^138^ The cavity loading system is dependent upon diffusion controlled by the shells, which provides release of the drug. They conclude that further research and understanding can assist in rational designing of nanocarriers for DDSs.^139^ The prime function of nanocarriers is to encapsulate the drug within itself until it reaches the tumor site following controlled drug release. The method used to understand this process of drug release is through dialysis.^140^ This technique however cannot be applied to hydrophobic nanocarrier drug loaded substances. Thus, to overcome such a problem Bouchaala et al.^141^ developed a novel technique for the quantification of release of fluorescent moieties from LNCs using fluorescence correlation spectroscopy (FCS). For this study, LNCs that were nanoemulsion droplets were encapsulated by the hydrophobic Nile red derivative NR668.^141^ The study showed that sharp contrast classical FCS parameters are more effective in drug release compared to less bright light.^141^ Therefore, the researchers made use of the standard deviation of fluorescence fluctuations for analysis of release of the dye from the nanocarriers quantitatively. The drug release was found to be temperature dependent, and the rate declined at 37 °C after a 6-h duration of 50% delivery.^141^

**Figure 7:**
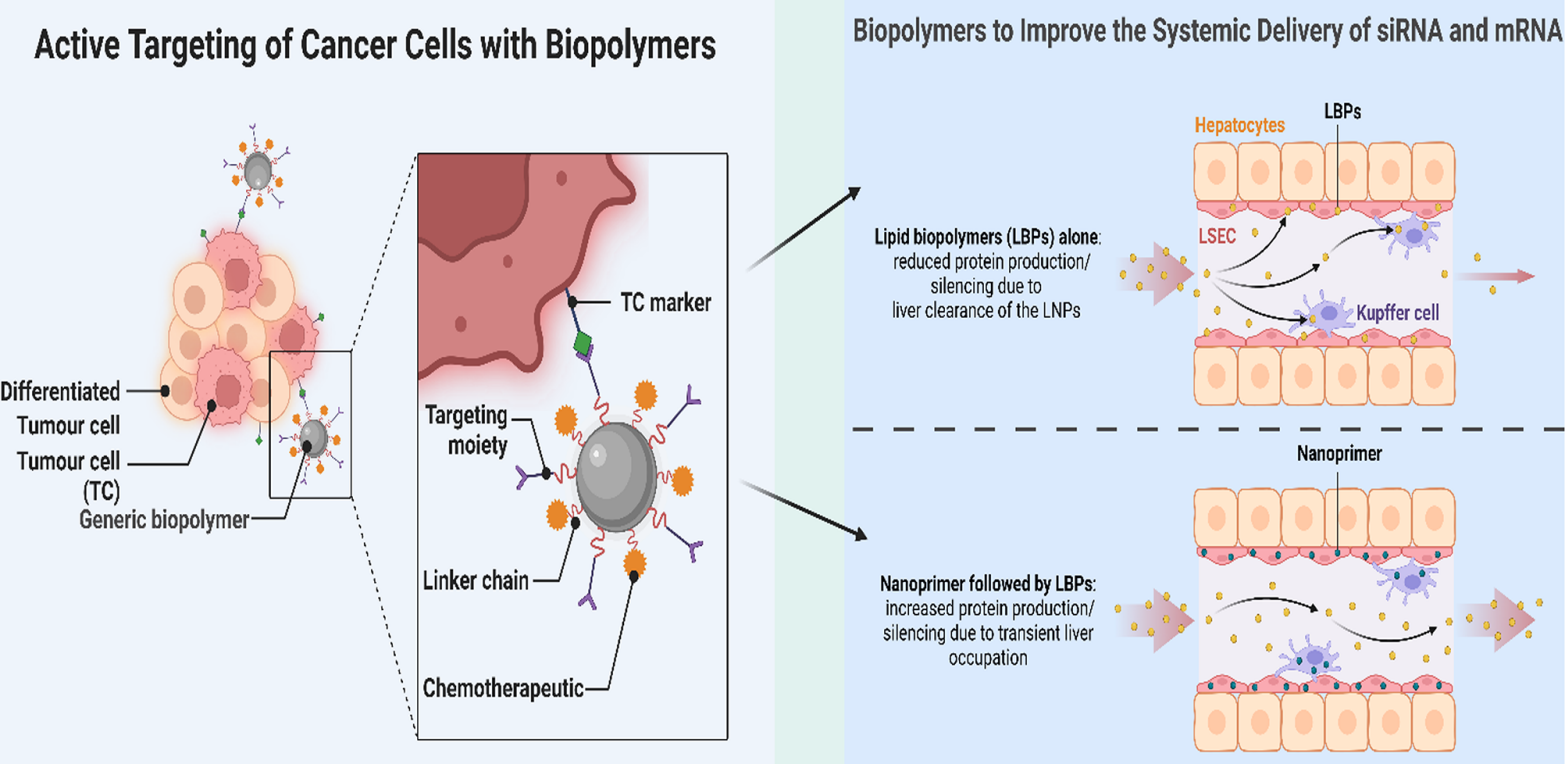
Active targeting of tumor cells in the application of biopolymers with proper delivery of siRNA and mRNA.

Similarly, a focused effort on newer techniques like the production of nanocrystals in a confined environment can be achieved within microfluidics channels and was reported by Fontana et al.^142^ We have previously cited that modified peptide moieties can serve as good nanocarriers for DDSs. In the latest work by Medea Neek et al.,^143^ researchers found that the peptide alone fails to act as a nanocarrier at the targeted tumor site. They concluded that the peptides could only function effectively as tumoricidal to the target cells, if that embedded in a caged protein which has virus-like structures.^143^ Moreover, the interaction of these virus-like structures and cage protein with the immune system has concluded that they have a better efficacy as a DDS^143^ shown in Figure 7.

## 4 Intracellular Trafficking of Biopolymer-Based Nanoparticles in Cancer Cells

The process of intracellular trafficking of biopolymer-based nanoparticles by cancer cells is a complex and dynamic phenomenon that holds significant importance in the targeted delivery of therapeutic drugs and imaging agents to specific locations within cancer cells. The complex process entails intricate interactions between nanoparticles and diverse biological components, which play a crucial role in determining the efficacy of cancer treatment. Biopolymer-based nanoparticles, which are generated from polymers such as poly(lactic-*co*-glycolic acid) (PLGA), chitosan, or polydopamine (PDA), offer distinct benefits such as biocompatibility, adjustable characteristics, and the capacity for functionalization. The aforementioned attributes make them highly desirable candidates for the purpose of cancer therapy since they enhance the effectiveness of medication delivery and imaging techniques.

The transportation of biopolymer-based nanoparticles within cancer cells occurs through a series of consequential phases:

(a) **Cellular uptake:** The primary phase focuses on the uptake of nanoparticles by cancer cells. The process under consideration involves various processes, including endocytosis (such as clathrin-mediated endocytosis, caveolae-mediated endocytosis, and macropinocytosis) and phagocytosis. The precise mechanism of absorption is contingent upon various elements, including the size of the nanoparticles, their surface charge, and the process of functionalization. The nanoparticles have the potential to be modified in order to enhance the process of cellular uptake by including ligands that specifically bind to receptors that are excessively expressed on the surfaces of cancer cells.
(b) **Endosomal escape:** Following internalization, nanoparticles frequently become sequestered within endosomes or lysosomes, leading to a restricted environment. In order for nanoparticles to achieve their desired therapeutic or imaging effects, it is imperative that they successfully evade these compartments and gain entry into the cytoplasm. Specific biopolymer-based nanoparticles are intentionally engineered to react to the acidic environment found in endosomes, thereby triggering the discharge of their payload into the cytoplasm. This particular phase holds significant importance in preventing the breakdown of cargo within lysosomes.
(c) **Intracellular trafficking:** Once nanoparticles have evaded endosomes, they proceed to navigate within the cytoplasm, progressing along a sequence of intracellular trafficking routes. The aforementioned pathways encompass engagements with microtubules, molecular motors, and other constituents of the cytoskeleton. The use of cellular pathways can be leveraged in the design of nanoparticles composed of biopolymers, resulting in an increased ability to move within the cell.
(d) **Nuclear localization:** In certain circumstances, nanoparticles are designed to specifically target the cell nucleus, serving purposes such as imaging or gene delivery therapy. This requires the surmounting of other obstacles, such as the nuclear envelope. The introduction of surface changes and the use of specific cargo molecules can enhance the nuclear entrance of nanoparticles, hence providing precise and targeted functionalities.
(e) **Cargo delivery and release:** Once biopolymer-based nanoparticles have reached their designated subcellular locations, it is crucial for them to effectively transport their cargo, which may include therapeutic medicines, imaging agents, or genetic material. Controlled release techniques can be customized according to characteristics such as pH sensitivity, enzymatic breakdown, or external stimuli such as light or heat.
(f) **Interaction with subcellular organelles:** The involvement of nanoparticles in cellular processes may require interactions with different subcellular organelles, such as mitochondria or the endoplasmic reticulum. These interactions exert a significant impact on cellular processes and, as a result, have implications for therapeutic outcomes.
(g) **Biodegradation and clearance:** These are crucial aspects of the cellular fate of biopolymer-based nanoparticles, as they contribute to the nanoparticles’ safety and biocompatibility. As the nanoparticles navigate the complex environment within the cell, a natural process of biodegradation occurs over time, leading to the slow breakdown of the nanoparticles. The capacity of biopolymer-based nanoparticles to undergo degradation over time is a noteworthy characteristic that distinguishes them, thereby reducing the potential risks associated with extended accumulation inside the cellular milieu. The relevance of biodegradation is rooted in its substantial consequences for both the efficacy of therapeutic interventions and the long-term safety of such interventions. The progressive disintegration of the nanoparticles into smaller components facilitates a synergistic alignment between the therapeutic efficacy and cellular uptake. Upon the disintegration of nanoparticles, they undergo a transformation into metabolites and fragments, which possess a higher degree of assimilation and can be more efficiently digested by the cellular machinery. The deliberate coordination of this change guarantees that the nanoparticles do not persist indefinitely within the cellular boundaries, reducing the probability of any detrimental consequences arising from prolonged nanoparticle existence.
(h) **Cellular responses:** The complex interaction between biopolymer-based nanoparticles and cancer cells elicits a wide range of significant cellular responses, including intricate changes in gene expression, modification of signaling pathways, and dynamic immunological reactions. The inclusion of nanoparticles in the cellular milieu initiates a series of interconnected processes that play a crucial role in defining these sophisticated therapeutic agents’ overall therapeutic effectiveness and safety. Gaining a thorough comprehension of these complex and diverse reactions is crucial, as it forms the fundamental basis for enhancing and optimizing the utilization of these nanoparticles in cancer treatment.
(i) **Immune reactions and immunomodulation:** In addition to their direct effects on cancer cells, nanoparticles made from biopolymers also engage with the immune system, resulting in a diverse range of immunological reactions. The ability of nanoparticles to activate various immune cells, including dendritic cells, macrophages, and T cells, has the potential to elicit an immunological reaction against cancerous cells. The capacity of nanoparticles to regulate immune responses presents opportunities for synergistic combination therapy, wherein the immune system is utilized to enhance the effects mediated by nanoparticles. On the other hand, nanoparticles can suppress immunological responses to prevent excessive inflammation, thus underscoring their potential for immunomodulation.

## 5 Theragnostic Application of Biopolymer for Anticancer Therapy

### As Anticancer Agents

Due to the large number of fatalities caused due to cancer, it is necessary to identify and develop therapeutic agents that are efficacious and have less side effects.^144^ Research conducted on chemotherapeutic agents shows many enhancements and modifications. However, they still possess detrimental effects such as harming of normal cells as well as poor delivery to the target site.^145−148^ Starch, among other polymers, has been incorporated into nanodrug formulations with anticancer agents due to many advantages such as its abundance in nature, non-cytotoxicity, biocompatibility, biodegradability, non-immunogenicity, stability in air, as well as compatibility with most drugs.^149^Table 3 provides some examples of biopolymer-based nanocarriers used as anticancer drug delivery agents.

**Table 3:** Biopolymer-Based Nanocarrier Targeting Tumor/Cancer Cell Lines

**Nanocarrier**	**Dimension**	**Drug moieties**	**Studies**	**Cancer cell**	**References**
Propyl starch	229 nm	Docetaxel	*In vitro*	Colon cancer	(150)
Amine functionalized polyacrylamide	39.4–46.3 nm	Photosensitized drug (Photofrin conjugated)	*In vivo*	Breast cancer	(154)
RGD peptide (arginine-glycine-aspartic acid) conjugated triblock polymer	99.8 ± 17.9 nm	Camptothecin	*In vitro*/*in vivo*	Brain cancer/glioma	(174)
Amine modified layered double hydroxide		NIR optical contrast agent, Indocyanine green	*In vitro*/*in vivo*	Colon cancer	(187)
Chitosan	9.4–23.4 nm	Quantum dots	*In vitro*	Non-Hodgkin lymphoma cancer cells	(198)
PEG functionalized melanin nanoparticles	7.5 nm	Image contrast agents	*In vivo*	Hepatocellular carcinoma	(205)

Prajakta Dandekar et al.^150^ synthesized a hydrophobic starch derivative named propyl-starch to understand its application in NP formulations that encapsulated anticancer agent docetaxel using the solvent emulsification diffusion technique.^150^ The researchers observed an enhancement in efficacy and availability of the drug in the cancer cell line due to its “nano size”. Moreover, cytotoxicity was also not observed in *in vivo* studies and perinuclear localization of the NPs also confirmed its targeted association with the nucleus (peri/intra).^150^

**Figure 8:**
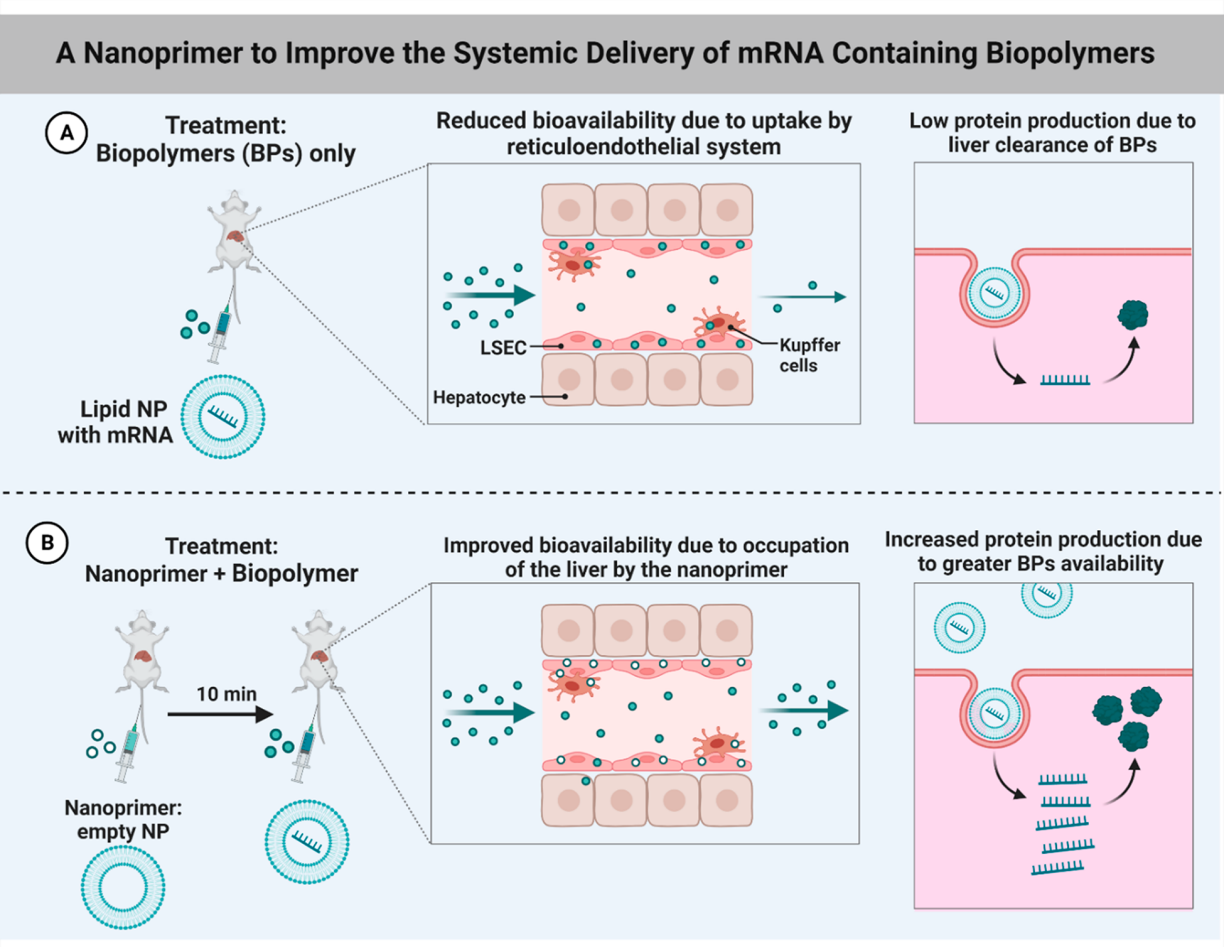
mRNA modified biopolymer-based assisted drug delivery: (A) lipid NP with mRNA method and (B) nanoprimer + biopolymer approch.

Proteins like gliadin, soya, bovine serum albumin (BSA), milk protein, zein, elastin, and gelatin show efficient drug delivery characteristics at a nano level. Protein-based polymers have also been used by researchers as anticancer and antitumor delivery agents.^151^ EPR has been proved in many animal models and was also discussed by Taurin et al.^160^ Nanomedicines showed effectiveness and accumulation at the tumor site up to 27-fold more than normal drug even after 6 h of administration,^152^ as shown in Figure 8. In general, besides biocompatibility and biodegradability, protein NPs offer an advantage in synthesis as they can be prepared under mild conditions without harmful chemicals either through a coacervation/dissolvation technique, emulsion solvent extraction technique, or complex coacervation techniques.^153^

Shouyan Wang et al.^154^ extensively researched biodegradable polyacrylamide NPs with an amine functionality as a cancer theragnostic and included studies based on active cancer cell targeting, imaging via fluorescence, and photodynamic therapy.^154^ The nanodrug development involved the addition of primary amino moieties and cross-linkers that were biodegradable during the polymerization process of NPs along with the introduction of photodynamic and fluorescent agents in the NP matrix that were conjugated with PEG and ligands of tumor on the surface.^154^ Studies performed *in vitro* on human breast cancer cells resulted in efficient drug delivery to the site. In conclusion, the benefits of polymer-based NPs as nanocarriers for tumor theragnostics that are biodegradable and multifunctional were demonstrated in the study.^154^ According to Lohcharoenkal et al.,^155^ proteins are much safer as nanocarriers of anticancer agents. An albumin-bound nanocarrier with a particle size distribution of 130 nm showed effectiveness as an anticancer drug.^155^ Their study also revealed the FDA approval of albumin-bound paclitaxel (Abraham, ABI-008) for metastatic breast cancer which exemplifies the clinical feasibility of this approach.^155^ When subjected to high pressurized homogenization, albumin’s dimensions are 100–200 nm which is effective in loading and delivering paclitaxel to the site.^155^ The study also revealed that cationic bovine serum albumin (CBSA) can be used as a new siRNA delivery system for the treatment of metastatic lung cancer. Milk protein casein NPs that encapsulated doxorubicin showed very interesting results in oral delivery, as it was highly effective in hepatocellular carcinoma treatment. The overview also helped to understand the various approaches undertaken for the development of protein-based anticancer therapeutics and their mechanisms.^155^

Polydopamine (PDA) has received considerable attention in the scientific community due to its remarkable characteristics, including biocompatibility, facile production, strong near-infrared absorption, high photothermal conversion efficiency, and efficient binding of metal ions. Extensive research has been conducted on multifunctional nanosystems containing programmable logic devices (PDA), primarily for biomedical applications, due to the notable characteristics exhibited by these systems. A thorough examination is essential to delineate the synthetic methodologies employed in producing diverse nanoparticles (NPs) incorporating polymeric drug carriers (PDA) for advanced cancer diagnosis and treatment. It also examines the diverse applications of these NPs in different imaging modalities and their potential therapeutic implications in cancer treatment.^156^ The genesis of the PDA (polydopamine) phenomenon can be traced back to the adhesive properties exhibited by mussel adhesive proteins when they interact with solid surfaces. The versatile substance known as polydopamine (PDA) was first produced by Messersmith et al. by the process of auto-oxidative polymerization of dopamine (DA) in mildly alkaline circumstances.^157^ Although the precise mechanism is not yet fully understood, the distinctive characteristics of PDA have led to its use in several domains such as healthcare, renewable energy, and environmental sciences.^148^ The utilization of PDA exhibits desirable characteristics, including a preparation process that is both mild and solvent-free, the attainment of homogeneous particle size, and a wide range of optical absorption. The utilization of this nanoplatform for tumor imaging has been observed to be endogenous, particularly in the context of photoacoustic and fluorescence imaging techniques.^158^ The significance of this issue lies in the limitations of conventional tumor detection procedures, such as biopsies, which lack both specificity and sensitivity. Additionally, imaging techniques are not only expensive but also subject to certain restrictions. PDA-based nanoplatforms have been found to provide a noninvasive and highly effective means of diagnosing tumors (10.1038/nature06917). In addition, the ability of PDA to interact with metal ions such as Cu^2+^, Fe^2+^/Fe^3+^, Mn^2+^, and Zn^2+^ through its catechol and amino groups allows for its use in several medical imaging techniques, including magnetic resonance imaging (MRI), computed tomography (CT), and positron emission tomography (PET).^149,159^ The capacity of PDA to effectively encapsulate medicinal or contrast materials enhances its efficacy as a delivery mechanism for imaging-guided therapy. The surface-modifying properties, biocompatibility, and biodegradability of PDA contribute to its increased suitability for many applications. It is worth mentioning that polydopamine (PDA) demonstrates the ability to convert light into heat and scavenge reactive oxygen species (ROS), rendering it highly useful in wound healing and cancer therapy. Therefore, the multifarious features of PDA have stimulated substantial research in the field of biomedical applications, including cancer diagnosis and therapy.^41^ The ease of synthesis, adaptability in surface modification, imaging capabilities, and potential for therapeutic applications of nanosystems containing polydopamine (PDA) render them highly promising instruments in the field of biomedicine.

Polymer conjugates, liposomes, micelles, and metal NPs have also been used as nanomedicines for anticancer therapeutics. Taurin et al.^160^ studied enhanced permeability rate (EPR) and effectiveness of such nanomedicines on tumor cells. They listed advantages and drawbacks of the EPR in their report. According to their view, tumors consist of defective endothelial tissue with dimensions between 300 and 4700 nm. They also lacked a smooth muscle layer or innervations and a wide lumen, and they possessed impaired and nonfunctional receptors for angiotensin II.^160^ They also observed that the nanomedicine accumulation in the target cell is dependent on factors such as biodistribution, circulation time, tumor uptake, bradykinin, nitric oxide (NO), prostaglandins, peroxynitrite, and matrix metalloproteinases.^160^ Variations between normal and tumor tissue were observed with respect to nanomedicine targeting owing to the enhanced permeability and retention (EPR) effect.^161^ This occurrence is expected since normal tissue contains endothelial cells that are tightly connected thereby preventing the diffusion of nanomedicine to the exterior of the blood vessel, while tumor tissue has large openings or fenestrations between the endothelial cells thus allowing nanomedicines to reach the matrix and the tumor cells through the EPR effect.^162^ VEGF secretion by tumor cells, stroma cells, and macrophages causes an enhancement in permeability and stimulates angiogenesis and endothelial cell migration toward the tumor.^160^ However, a considerable proportion of nanomedicine does not reach the targeted tumor area due to entrapment like, nonspecific interaction with matrix, matrix-composed collagen, or removal through macrophage endocytosis.^38^ These nanomedicines tend to accumulate and concentrate in the periphery of the tumor, while only a small proportion will diffuse to the tumor’s center. They also stated that NPs are easily identified by the body as foreign particles and, as a result, undergo a rapid uptake and elimination by specialized cells belonging to the reticulo-endothelial system (RES).^163^ To improve cellular uptake, uniform coating with bioligands could be performed as per the reviewer’s view. It is well-known that polymer conjugates behave as covalent ligands and therefore release of the drug is based on their bond structure and on factors such as pH, acidic pH of lysosome, temperature, or enzymatic cleavage.^164^

**Figure 9:**
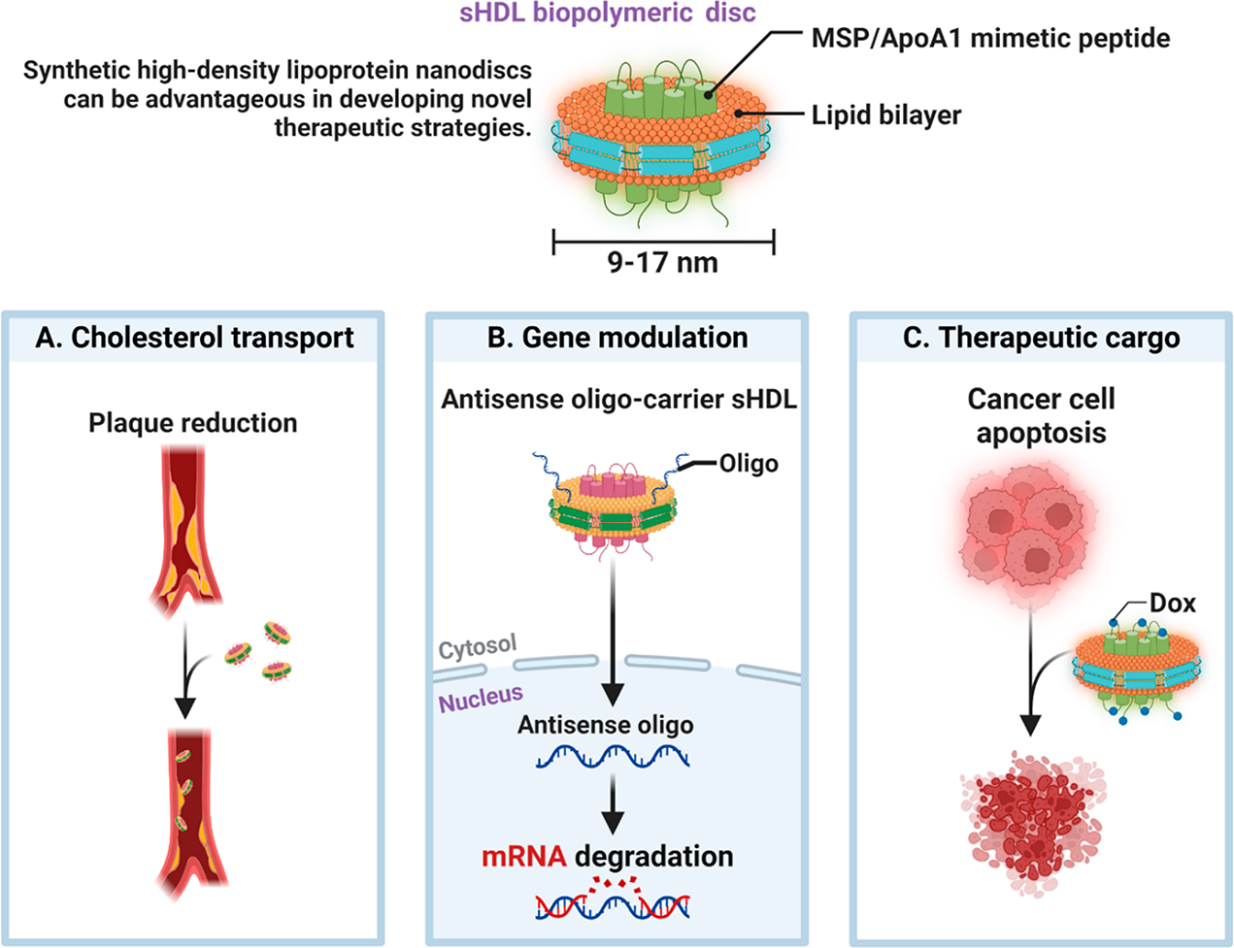
Biopolymer-based nanocarriers (9–17 nm) are hybrid and contain a combination of lipids as well as genes like (A) cholesterol, (B) gene, and (C) other therapeutic cargo.

The lipid, liposomes, and micelles^165^ were also found to take part actively as anticancer agents to target breast cancer. Breast cancer patients prefer an oral drug administration route, according to Andey et al.^166^ A study was performed to observe the oral efficacy of lipid conjugated estrogenic derivative (ESC8) loaded with the anticancer agent cisplatin in a NU/NU xenografted mice model.^166^ It was observed that when ESC8 was administered alone it cured 74% of the breast cancer cells but when cisplatin was loaded with ESC8 the percentage cure increased to 87%.^166^ Indocyanine (IC)_10_ was also incorporated, which resulted in effective imaging analysis when *in vivo* studies were performed. Miao et al.^167^ reported the cellular mechanisms and treatment for the resistance of anticancer drugs using dual nanomedicines from liposomes, lipid nanocarriers, micelles, polymer conjugates, lipid coated drug loaded calcium phosphate, and combinations of siRNA chemotherapeutics in single nanocarriers.^167^ The dual functionality of drug liposomes in treatment of resistant cancers was extensively studied by Mu et al.^168^ These described the drug as possessing phospholipid biliary vesicles of dual functionality: (1) basic therapeutic efficacy of drug and (2) extended effect of the drug carrier.^168^ As a result, this dual functional liposome can therefore eliminate drug resistance observed in cancer through the circumvention of the efflux of the drug produced by the adenosine triphosphate binding cassette (ABC) transporters, elimination of cancer stem cells, destruction of mitochondria, initiation of apoptosis, regulation of autophagy, destruction of supply channels, utilization of the microenvironment, as well as genetic silencing of cancer cells.^168^ Mitochondrial targeting by dual-function drug liposomes and apoptosis induction has also been observed in drug resistant cancer. As mentioned earlier, lipid nanocarriers have good efficiency for delivering the drug at the tumor site.^168^ Recent studies dealt with understanding the molecular basis of epithelial ovarian cancer and its treatment. In this regard, Bondi et al.^169^ performed research on the development of curcumin-based NPs entrapped in lipid nanocarriers. The nanosystem helped in overcoming the drawback of poor bioavailability and enhanced the antitumoral activity of curcumin.^169^ In the study, three combinations of curcumin-loaded lipid nanocarriers were developed, including (1) compritol–captex, (2) compritol–miglyol, and (3) compritol NLCs. Dissolution of curcumin was done in the hot lipid phase followed by high-speed homogenization at 13000 rpm for 10 min further followed by lyophilization.^169^ Experiments performed *in vitro* using cisplatin sensitive (A2780S) and resistant (A2780CP) EOC cells showed that curcumin loaded into NLCs provided therapeutic efficacy that was comparable to that of free curcumin as assessed by cell viability.^169^ Moreover, in both of these cell lines, curcumin loaded NLCs showed an enhanced anticancer potential in clonogenicity assays as compared to free curcumin. So far, the works cited portrayed the usage of lipids and polymers as nanocarriers.^169^ According to Date et al.,^170^ lipids are cost-effective but have limitations such as instability, a burst release of the drug, and limited surface functionalization. In contrast, polymeric systems offer diverse chemical modifications and enhanced stability along with release of drug in a controlled manner.^170^ Conversely, the inability to scale up and limited drug loading capacities limit their use. Nanocarriers that are hybrid and contain a combination of lipids and polymers overcame these disadvantages and maintained the advantages of both systems^170^ shown in Figure 9. Date et al. also presented a description of the mechanism involved in drug delivery of lipid polymer hybrid nanomedicine formulations both *in vivo* and *in vitro*.^170^

Nowadays, combination therapy has become an essential strategy for cancer treatment. It is due to the short half-lives of chemotherapeutic drugs. Dai et al.^171^ focused on dual nanomedicine combination therapy wherein a combination of therapeutic strategies between various nanomedicines or drug-loaded nanocarriers was focused on rather than the codelivery of different drugs via a single nanocarrier.^171^ The present treatment strategies using combination therapy are based on targeted nanomedicines. These strategies can be grouped into two major categories: (1) combination therapy based on tumor cell killing and (2) combination therapy based on tumor cell targeting.^172^ The schematic illustration describes the nanomedicines targeting the same or sometimes different subtypes of tumor cells. As the nanomedicines targeting tumor cells with multidrug resistance (MDR), tumor stem cells, and extracellular matrix (ECM), thus a high chance to kill all the cancer cells.

Nanomedicines showed extravagant properties in active or passive targeting of tumor cells in cancer therapy.^173^ However, most nanomedicines had several drawbacks such as the suboptimal targeting effect and leakage of the drug, which resulted in an unsatisfactory treatment outcome. Therefore, to overcome this situation, Wang et al.^174^ developed a hierarchical responsive nanomedicine (HRNM) for the programmed delivery of chemotherapeutics using cyclic Arg-Gly-Asp (RGD) peptide conjugated with a triblock copolymer such as poly(2-(hexamethyleneimino)ethyl methacrylate)-poly(oligo-(ethylene glycol)monomethyl ether methacrylate)-poly[reduction-responsive camptothecin (PC7A-POEG-PssCPT) through self-assembly.^174^ This study revealed the effective passive targeting of HRNM to the tumor site. The *in vitro* and *in vivo* studies showed antitumor activity with reduced normal cytotoxicity.^174^

Here, we present very recent and interesting research works besides the traditional methods followed and provide concepts of some newer approaches initiated by several groups of researchers. Yi Li et al.^175^ reported the ability of charge convertible polymers as nanocarriers for disease cure. Polymers that are negatively or neutrally charged become active at the tumor site, disabling cancer cells and increasing the viability of normal cells. Joint research work of scientists from India and USA presented by Padmakumar et al.^176^ showed some remarkable observations in late-stage ovarian cancer cells wherein anticancer agents were delivered at the tumor site by nanocarriers formulated by nanotextile implants woven through the electrospinning of biopolymeric materials. The recent developments on enzymes at the nanoscale also attracted great attention as a drug delivery system. Dheer et al.^177^ pointed out some remarkable features of the cathepsin-based nanodrugs and their mechanism in anticancer treatment.

The review already focused on the types of biopolymers and their nano range utility in drug design. Nanopolymers also serve as nanocarriers for cancer therapy. Dong et al.^178^ extensively surveyed new techniques of nanocarrier formulation for the effective tumoricidal activity. They reported various techniques of nanoparticle formulation which includes techniques like ionic stabilization and steric stabilization, use of polymeric ligands and small-molecule ligands, phase transfer (PT), the ligand exchange and ligand addition effect, and coupling strategies for bio functionalization.^178^

They even focused on magnetism-induced anticancer NPs and magnetic thermal NPs for drug delivery. According to the review, the release of heat by magnetic NPs, in the presence of a high frequency magnetic field, causes the apoptosis of the cancer cell without affecting normal cells. Furthermore, they described stimuli sensitive polymers and zwitterionic polymers which, following entry into cells of the tumor, the positive and negative charge balance will be broken bringing about changes in conformation, drug release in cells of tumors, and near infrared (NIR) sensitized smart ligand antibody interaction. Figure 7 depicts the 3 mechanisms, (i) a penetration enhancer targeting the moiety of interest, (ii) apoptosis due to the antitumor drug, and (iii) through utilization of a carrier comprised of lipids, metals, and ceramics in NPs. The study also revealed the usage of photoresponsive materials that are NIR-light-based and various other target-based receptors for cancer therapy. They also focused on modification of the surface of the NPs as well as the active and passive targeting mechanism.

### In PDT

Chemotherapeutic drugs have shown difficulty releasing the drug at the tumor site and have been found to be harmful to normal healthy cells.^179^ Radiation therapy was also shown to be detrimental to healthy cells, and therefore, to bypass these limitations, a new theory named photodynamic theory (PDT) has emerged recently.^180^ This theory involves utilizing components such as a photosensitizer, drug-activating light at a specific wavelength, and oxygen.^181^ Photosensitizer light brings about energy generation thus causing development and formation of ROS which results in death of the cancerous cells.^182^

Porphyrin is one such promising sensitizer being used so far along with a chlorine-based nanoformulation to enhance the EPR of nanomedicines at the tumor site. Porphyrin has been loaded with polymeric NP, magnetized NP, silicon particles, and liposomes.^183^ Fluorescence NPs have served extensively in targeting breast, lung, and hepatic carcinoma according to Alyssa Master et al.^184^ The study also reveals that chlorinated NPs loaded with chitosan NP and human serum albumin worked efficiently. Other photosensitizers, namely, phycocyanin, indocyanine, hypericin, and methylene blue, have worked brilliantly when loaded with several NPs of polymeric conjugates leading to apoptosis of cancer cells.^184^ Nanoparticles have provided various methods to enhance the photosensitizer delivery of the targeted-tumor component through optimum encapsulation of the photosensitizer, drug inactivation protection that is induced by plasma, premature leakage of the drug, an enhancement in the uptake within the tumor tissue and cell along with specific drug release in the tumor environment and distribution.^184^

Indocyanine green (ICG), an amphiphilic tricarbocyanine dye, has been considered safe for use by the FDA. The emission maxima of ICG are around 800 nm; as a result, it is very suitable for bioimaging applications along with a high signal-to-background ratio.^185^ According to Sheng et al.,^186^ ICG combined with DoX loaded in nanoagents proved more stable and showed prolonged circulation in blood even in laser radiation.^186^ The study also revealed that *in vivo* biodistribution of ICG containing NPs with 50 nm particulate size can be used to target the liver.^186^ Moreover, PDT of ICG combined with poly(propargyl acrylate) NPs coupled with radiation of 780 nm decreased tumor cell growth significantly and statistically. The review also mentioned the use of modified ICG with BSA along with core magnetic nanoparticles for high optical imaging performance in DDSs.^186^

According to Pei Wei et al.,^187^ an amine modified into a layered double dihydroxide (LDH) along with indocyanine green as a contrast agent was declared safe for usage by the FDA for optical imaging in *in vivo* studies. LDH showed better biocompatibility and non-cytotoxicity. It also enhanced the solubility of drug. LDH–NH_2_–ICG coated chitosan confirmed the stability of nanocomposites in the *in vivo* studies.^187^ Furthermore, the noninvasive *in vitro* imaging studies of LDHs–NH_2_–ICG nanoparticles coupled with varying amounts of coatings of chitosan which were administered intravenously in nude mice that were anesthetized showed a visible fluorescence in the liver and lungs. The pairing of chitosan and LDHs–NH_2_–ICG showed an enhanced potential for the development of organ-specific DDSs and *in vivo* contrast agents for cancer diagnosis and chemotherapy.^187^

Nowadays PDT is very effective in cancer therapy, and palladium NPs are not only cost-effective but also provide photoacoustic imaging and photothermal therapy.^188^ Phan^189^ designed a porous flower-shaped palladium nanostructure using chitosan vitamin C, which, when administered *in vitro*, the photons absorbed by the nanostructure convert into heat energy, thus destroying cancer cells.^189^ In a similar investigation, sugar-based moieties due to their richness in hydroxyl protons can be used for phototherapy in cancers as per Han et al.^190^ These moieties make use of novel MRI technology and chemical exchange saturation transfer (CEST), therefore preventing the need of chemical labeling.^190^

Jin et al.^191^ reported that, though Au NPs attained great importance in cancer phototherapy, they have a limitation of surface toxicity. To reduce this, Au NPs need to be coated with organic polymers.^191^ There are advantages and disadvantages of organic polymer coating on gold nanorods; thus, further modification is essential to coating gold nanorods with polymer.^191^ Conjugation with target molecules could be done that can enhance the photothermal therapy (PTT). Combination therapy like chemotherapy, gene therapy, PTT, and the organic polymeric coating would enhance the DDS of gold nanorods.^191^

The use of PDT has now become an emerging trend in cancer therapy. Over the years, the revolutionary technologies of PDT have shifted sources moving from ultraviolet/visible (UV/vis) light to near-infrared (NIR) light up conversion fluorescence to NIR two-photon excitation to X-ray radiation and finally self-illumination (chemiluminescence, bioluminescence, and Cerenkov luminescence).^192^ This fact has brought about the promising development of PDT that offers better therapeutic efficacy. The use of these technologies, however, offers drawbacks. These include injury caused due to ionizing radiation, ablation of tissue which occurs due to long-term irradiation, and insufficient self-illumination efficiency.^193^ Therefore, the use of persistence luminescence has become important to eradicate these problems. The material of persistent luminescence (PersLum) can emit fluorescence even after the light source has ceased.^194^ Sun et al.^195^ developed a PersLum calcium alginate-based hydrogel for highly efficient PDT. This hydrogel possesses favorable characteristics such as biological compatibility, persistent luminescence which is bright, renewability of red light, acceptable syringe ability along with a strong fixing ability in cancers which can be introduced easily as a powerful localized light source via injection *in vivo* for superior persistent luminescence-sensitized photodynamic therapy.^195^

### In Cancer Imaging

Despite the innovative research conducted in the field of cancer, it has taken millions of lives around the globe, especially in developing countries. Early stage cancer detection has now become one of the main focuses of research in the field of nanomedicines by scientists.^196^ The nanomaterial semiconductor known as quantum dots was found to be very potent in the detection of cancer when bound to biological molecule as it is very effective in the optical imaging of necrotic cells.^197^

Mansur et al.^198^ designed, synthesized, and characterized new multifunctional immunoconjugates comprised of a fluorescent inorganic core containing quantum dots (QDs) along with an organic shell composed of antibody-modified polysaccharide chitosan and applied them for the *in vitro* diagnosis of cancers such as non-Hodgkin lymphoma (NHL).^198^ The images of transmission electron microscopy (TEM) and UV–vis absorption results showed the development of ultrasmall nanocrystals whose average diameters ranged between 2.5 and 3.0 nm. Results of photoluminescence also showed that the immunoconjugates produced a “green” fluorescence under ultraviolet excitation. The laser light scattering immunoassay also confirmed binding of antigen to the quantum dots, thereby proving to be promising for the early stage detection of carcinoma.^198^

The glycol chitosan has been one of the most interesting target substances that have gained attention as a theragnostic agent.^199^ According to Rhee et al.,^199^ when glycol chitosan is combined with siRNA, stability is observed *in vivo*. Fullerene, an essential luminescence agent, has proved ineffective when combined with a biological molecule.^199^ However, it was found to enhance optical imaging characteristics in detecting tumor cells when doped with glycol chitosan NPs.^199^ Similarly, porphyrins also obtained better results when glycol chitosan NPs were loaded with it. Photosensitizes are crucial for PTT as they absorb NIR followed by the conversion of luminous energy to thermal energy thereby producing cytotoxic heat to tumor cells.^199^ Since chitosan and other polymer conjugates can be incorporated with ICG and are deemed safe for use by the FDA, they are already being used as nanocarriers for PTT.^199^

In a similar investigation, Song et al.^200^ designed a new nanocarrier. The nanocarrier is comprised of a coating of chitosan near-infrared (NIR) layered double hydroxide–indocyanine green nanocomposites.^200^ This nanocarrier which contained a double layer of chitosan exhibited a more enhanced phototherapeutic effect when compared with uncoated LDH–NH_2_–ICG as well as when compared with LDH–NH_2_–ICG with a single layer chitosan coating due to the superior photosensitizer protection against photo and thermal degradations.^200^ ICG approved by the FDA is the most common contrast agent known for PDT and photoacoustic image guidance chemotherapy.^200^ However, the only drawback that ICG offers is poor blood retention, which has been found to cause loss of drug through the excreta of xenografted mice models. To stabilize ICG, a lot of research was conducted and it was observed that liposomal PLGA offers stability to ICG and thus remains for a prolonged duration in blood circulation.^200^ Wang et al.^201^ showed that PLGA ICG nanocarriers can also be used as potential nanocarriers as theranostic agents.

Park et al.^202^ observed some controversies in Förster energy resonance transfer (FRET) technology in DDSs. According to them the FRET inhibits luminescence from the gold nanoclusters thereby causing inefficiency in tumor image guidance chemotherapy.^202^ They therefore designed heat- as well as fluorescence-emitting gold nanoclusters that were loaded with albumin nanoparticles (Au NCs/Cy5.5-BSA-NPs), through the optimization of the quantity of gold nanoparticles, gold nanoclusters, and albumin taking into consideration interparticle distances.^202^ These gold clusters/BSA hybrid nanoparticles of dimensions of ∼150 nm displayed an acceptable hyper thermal effect in the presence of NIR light of 808 nm and possessed a well-preserved fluorescence intensity due to the contribution of the Cy 5.5’s surface modification. Au NCs/BSA-NPs significantly caused a suppression of the tumors and due to a decrease in FRET allowed visualization of tumor sites.^202^

Researchers found it challenging to study the interactions of nanoparticles with biological systems. As a result, control of the delivery systems *in vivo* is unpredictable. Donahue et al.^203^ proposed the thought of internalization of pathways and summarized the reasons for which the physicochemical properties of nanoparticles correspond and can affect cellular interactions.^203^

### As a Nanocarrier

**Figure 10:**
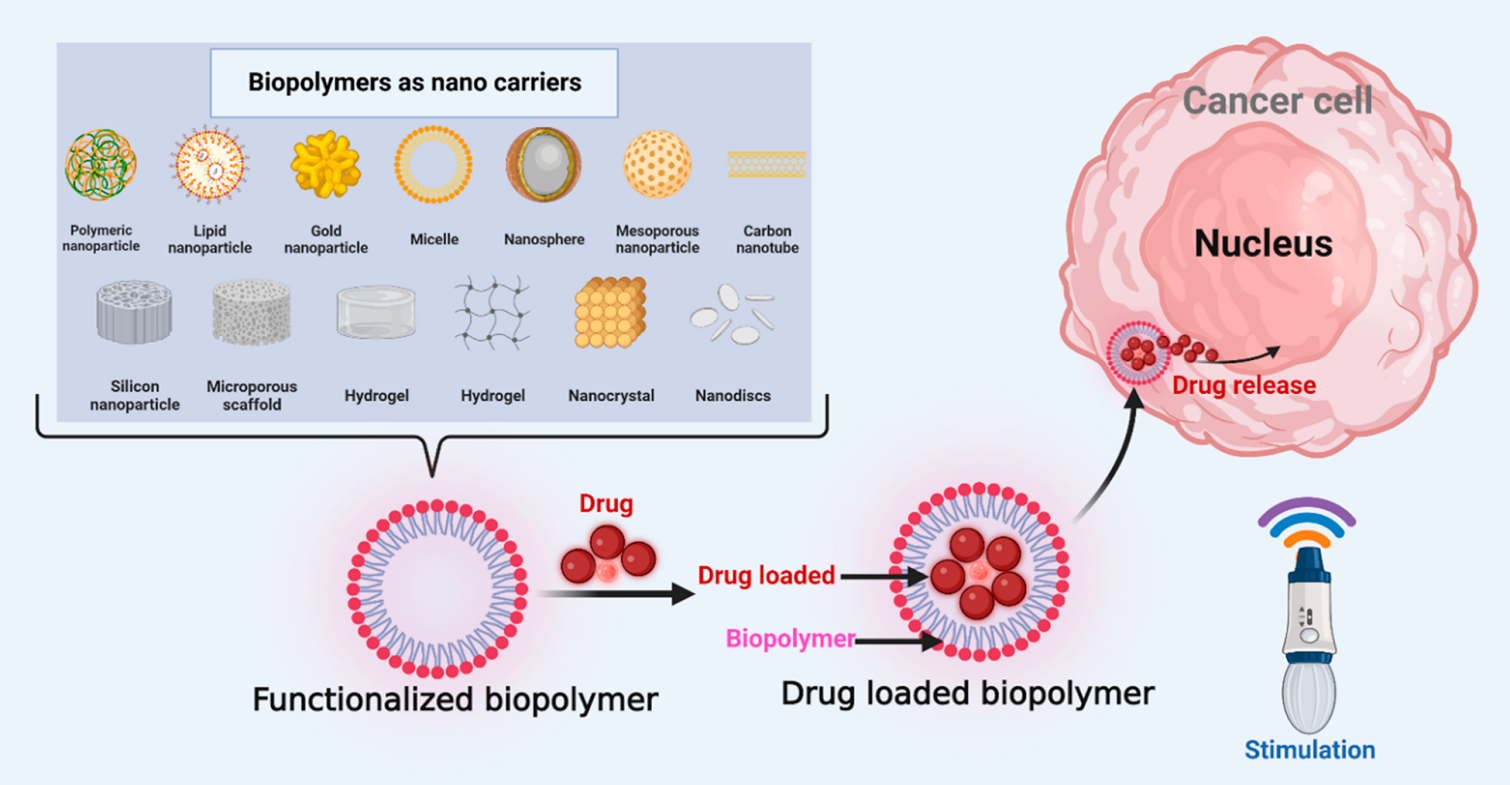
Nanoparticles work as functionalized nanocarriers with a combination of biopolymers.

Nanocarriers for image guidance have received tremendous attention as theragnostic agents. Polymeric materials and organic nanosystems have also been developed for PTT.^204^ Zhang et al.^205^ researched the ability of melanin NPs as efficient drug delivery systems for image guided chemotherapy.^205^ Thus, melanin is a biopolymer that possesses acceptable biocompatibility, biodegradability, and photoacoustic properties that are intrinsic in nature and the capability to bind to drugs which can thus be used in the development of an endogenous nano-DDS that is efficient for image-guided chemotherapy.^205^ NPs of melanin were formulated by loading them with the drug sorafenib to increase its hydrophilicity. Melanin–sorafenib shares π (pi) bond interactions.^205^ The SRFMNPs showed an equivalent anticancer effect as compared to the NPs that were polymeric, while the final loading dose that was used in the MNPs system was less (SRF 4 mg kg^–1^, one time every 2 days for MNPs vs SRF 3 mg kg^–1^, three times every 4 d for polymeric NPs), which shows that the MNP-based drug-delivery system was more advantageous and could at least provide the same antitumor effect which is seen with traditional polymeric-NP-based DDSs^205^ shown in Figure 10. Moreover, the traditional nanoplatform for imaging-guided therapy requires functionalization, and the introduction of contrast agents is complicated and can cause toxicity. MNP formulation involves a simple preparative procedure and is thus more appropriate to safe imaging guided therapy.^39^

Bovine serum albumin has been established as a nanocarrier for efficient drug delivery. Recent studies portray its significance in fluorescence studies as it is inexpensive, is sensitive, and can be used for noninvasive optical imaging in biomedical applications.^206^ Bovine serum albumin (BSA) alone is insignificant and does not have photoluminescence properties. Thus, Pan et al.^207^ researched a nano-DDS comprised of BSA doped with gold nanoclusters, iron NPs, and gold nanorods as fluorescence agents. Hence, gold nanorods and clusters are nontoxic, inert, and superior as fluorescent probes rather than photobleachable agents and can also show potential for imaging *in vivo* due to their emission as well as two-photon excitation that lie within the near-infrared (NIR) “biological window” between 650 and 900 nm.^207^ Disease detection imaging can be performed by magnetic resonance imaging (MRI). The combination loaded with drug DOX showed efficient delivery in hepatocarcinoma cells significantly leading to apoptosis, whereas the photoluminescence of the nanocarriers benefited in visualization of the delivery via utilization of fluorescence microscopy.^207^

The understanding of the fate of nanocarriers *in vivo* is limited. Zhao et al.^208^ presented a detailed survey on the fate of nanocarriers *in vivo*. The study reflected the relationship between the properties of NPs and that of drug accumulation at the tumor site as well as enhanced retention.^208^ It described that nanocarrier circulation depends on the size, shape, and surface charge. Nanomaterials with 30 nm dimension can permeate deeply in tumors, and spherically shaped particles have better penetration rather than rod shaped particles.^208^ The surface charge is also significant and is related with drug accumulation.^208^ The increase in surface charge densities that resulted in a decline in tumor accumulation was observed with negatively charged NPs, while higher charge density positively charged particles showed an enhanced accumulation in tumors.^208^ The authors attributed this observation to the fact that NPs that possessed higher positive charge density could depart from the interstitial space more efficiently and could be internalized by tumor cells along with the associated endothelium.^208^ The hydrophobicity effect is also very important in the DDS nanocarrier mechanism wherein hydrophobicity enhances plasma protein’s absorbance, thereby providing faster blood clearance.^209^

Chitosan with fucoidan gold nanorods working as a nanocarrier for PTT is now a fascinating area of research for cancer therapy.^210^ Manivasagan et al.^210^ studied the *in vitro* and *in vivo* efficacies of the nanocarrier. They observed that fucoidan gold nanorods encamped in chitosan (dimensions of 51.87 ± 3.03 nm), in a NIR absorbance after intravenous injection to breast cancer Balb/c mice model (MDA-MB-231 mice model) followed by irradiation for 5 min post injection and after 6 h post injection at 54.4 °C. The study revealed that after 20 days of the laser treatment total ablation of tumor cells was achieved.^210^

## 6 Challenges and Outlook

Despite recent advancements in the drug delivery system and the utilization of a wide variety of nanomaterials with appropriate properties, significant problems still persist in cancer management and treatment. The enormous progress made in delivering drugs over the last several years has led many people to believe that nanomaterials will alter the whole healthcare system. However, only a limited number of nanoformulations have entered into clinical trials due to the difficulty in designing effective cancer nanotherapeutics. The physicochemical features of nanomaterials strongly influence biocompatibility and toxicity in biological systems. Therefore, it is important to take precautions during the fabrication and characterization of the nano-biomaterials used in delivering medicine to reduce the risk of unintended toxicity to normal cells. Because of their interactions with biomolecules, nanocarriers may tend to accumulate into protein aggregates, which disrupt the normal operation of formulating nanomedicine and render those nanodrugs useless for preventing the proliferation of cancer cells. In addition, the pharmacological efficacy of nanomaterials may be affected by how they are stored. Concerns about nanomaterial-related side effects, which may or may not have an immediate impact or be perceptible, provide another difficulty in drug delivery. Nanocarriers have the potential to cause unintended toxicity in the treatment of cancer by interacting with cells in a way that is detrimental to the treatment’s efficacy. Drug release profiles have not been shown to correlate well between *in vitro* and *in vivo* settings, which is a significant unresolved issue. Although targeted drug delivery has shown promise in cancer therapy and diagnosis, there is still much to discover about analyzing events during drug interaction at the intercellular level and making predictions. Important data on the potential of nanoformulations as therapeutic medicines are gleaned through animal studies. Enormous research is needed in several areas, including the cost-effectiveness of anticancer medicinal molecules and the immunogenic effects of these drugs on humans and the environment. Another significant hurdle is the scaling-up of the production of nanomedicine or biopolymeric products for commercial use.

Out of many, one important concern is the ability of crossing the blood–brain barrier (BBB) as many chemotherapeutic drugs are unable or inefficient to cross the barrier and thus fail to bind to their respective target receptor. To overcome this challenge, scientists develop some nanocarrier molecules capable of integrating or carrying both hydrophilic and hydrophobic drug molecules and delivering it target-specifically by crossing the BBB. These nanocarriers are stable compounds having minimal toxicity and an immunogenic effect on our body, which are protected from being degraded enzymatically before reaching their target location.

Although biopolymers offer unique advantages, large-scale production remains a challenge. Ensuring consistent quality and reproducibility of biopolymer-based nanoparticles on a commercial scale requires robust manufacturing processes. Innovations in scalable production methods, process optimization, and quality control will be crucial to bridging the gap between laboratory research and industrial applications.

Lastly, one of the major challenges is to obtain approval from the FDA for the *in vivo* application of bio-nanocarriers. Since the FDA has not issued any standards for products containing nanomaterials, another major problem is the difficulty of getting these medicines approved for use. Currently, utilized parameters are taken exactly from bulk material specifications. Due to the fact that regulatory decisions on nanoformulated therapeutics are reliant on individual assessments of benefits and risks, evaluations are time-consuming and add delays to commercialization. The emergence of multifunctional nanoplatforms will also likely make getting regulatory approval more challenging.

## 7 Future Directions and Perspectives of Biopolymer-Based Cancer Formulations

The realm of cancer therapy is witnessing a paradigm shift with the advent of biopolymer-based formulations. The combination of biology and materials science has given rise to a class of adaptable materials that show great potential in transforming cancer treatment tactics. As we contemplate the future, a multitude of captivating avenues and viewpoints arise, exerting an influence on the course of biopolymer-based cancer formulations.

(a) Personalised Therapies: The field of cancer treatment is undergoing a transformation from a uniform approach to tailored therapies that accommodate the unique genetic characteristics and illness profiles of individual patients. Biopolymers provide a platform for the implementation of personalized medicine, enabling the customization of formulations to meet the specific requirements of individual patients. The customization of biopolymer-based formulations through the fine-tuning of characteristics such as nanoparticle size, surface qualities, and cargo payloads enables the optimization of drug delivery, the minimization of adverse effects, and the enhancement of therapeutic outcomes.
(b) Combination Therapies: Combination therapies have become increasingly prominent in the field of medicine due to their potential to synergistically combine multiple treatment modalities. These therapies are particularly valuable in overcoming drug resistance and improving the overall effectiveness of treatment. Biopolymer-based systems have the potential to enable the simultaneous administration of diverse agents, including chemotherapeutic medicines, immunomodulators, and gene treatments. These platforms have the capability to coordinate complex therapy sequences, facilitating the controlled release of various medicines at precise stages of cancer growth. The inherent versatility of biopolymers allows for the development of formulations that effectively enhance synergistic effects while simultaneously reducing unfavorable interactions.
(c) Targeting and Image Precision: The future of cancer therapy is contingent upon the attainment of precise targeting and imaging capabilities. Biopolymer-based nanoparticles possess the capability to be manipulated in such a way that they can bear ligands with a high degree of specificity toward cancer cell surface indicators. This enables them to facilitate targeted administration, hence mitigating the occurrence of off-target effects. Additionally, the functionalization of biopolymers with imaging agents offers the capability to monitor treatment responses in real time, hence facilitating the customization of therapy by physicians in a dynamic manner.
(d) Overcoming Biological Barriers: The successful distribution of drugs encounters obstacles presented by biological barriers, including the blood–brain barrier and the stromal barrier found in solid tumors. Biopolymers have the ability to be engineered in a manner that allows them to effectively overcome these obstacles. Biopolymer-based formulations have the potential to open up novel possibilities for treating previously inaccessible areas by manipulating the size, surface charge, and functional groups of engineered nanoparticles to overcome existing obstacles.
(e) Immunomodulation Strategies: The utilization of immunomodulation strategies to leverage the innate capabilities of the human immune system in the fight against cancer is an emerging and rapidly growing area of research. The utilization of biopolymer-based formulations presents an opportunity to effectively administer immunomodulatory drugs to immune cells or the tumor microenvironment. These formulations have the potential to be developed in order to regulate immune responses, modulate inflammation associated with tumors, and improve the effectiveness of immune checkpoint inhibitors, thus introducing a new age of immunotherapies.
(f) Continuous Drug Release: Continuous release of drugs is a persistent obstacle in the field of cancer therapy, particularly in the context of chronic ailments. Biopolymers possess the capability to be manipulated in a manner that enables the provision of continuous and regulated release of drugs, hence facilitating the maintenance of therapeutic concentrations for prolonged durations. The aforementioned feature holds significant relevance in the context of slow-growing tumors, as it reduces the necessity for frequent administration of medication and enhances patient adherence to treatment protocols.
(g) Biodegradability and Safety: The future of cancer formulations places significant emphasis on the biodegradability and safety aspects, with a particular focus on ensuring low occurrence of adverse effects. Biopolymers, frequently sourced from natural origins, possess inherent characteristics of biocompatibility and biodegradability. As scientific progress continues, it is anticipated that there will be a refinement of biopolymer compositions in order to minimize the likelihood of eliciting immune reactions and to enhance their effective elimination from the body.
(h) Clinical Translation and Regulatory Approval: The process of translating cancer formulations based on biopolymers from laboratory experiments to practical application involves successfully navigating the intricate realm of regulatory approvals and conducting clinical trials. In the coming years, there is a growing expectation for enhanced cooperation among researchers, doctors, and regulatory bodies. This collaborative effort aims to optimize the approval process and facilitate the timely accessibility of groundbreaking therapies for patients.
(i) Hybrid Approaches: In the future, it is possible that biopolymers might be combined with other modern technologies, such as nanomedicine, gene editing, and 3D printing, in order to develop new formulations. The integration of hybrid methodologies has the potential to facilitate the development of versatile platforms that effectively tackle multiple facets of cancer treatment, encompassing diagnostic procedures, imaging techniques, targeted drug administration, and tailored therapeutic interventions.

## 8 Conclusion

Nanomedicine for biomedical application has sought enormous attraction nowadays not only in the scientific forum but also among the public. The newest approach of design principles has been reported in an animal model but was not actuated or translated. The natures of EP and R and their dynamics need to be clarified in a better perspective in clinical settings. Several attempts to enhance EP and R were carried out in an artificial rodent tumor model, which does not depict human physiology or pathology in terms of transport or delivery. Therefore, scientists must place emphasis on this aspect. The studies on nanocarriers conducted as DDSs and performed *in vitro* and *in vivo* showed discrepancies in clinical trials. Therefore, nanoparticles should be used with due caution. Thus, instead of developing better or enhanced DDS target site drugs, researchers must give enormous importance in overcoming the adverse effects of existing nanomedicines, thereby widening the prospective for anticancer nanomedicines with better efficacy. In the past decades, researchers wanted to bridge the gap of the inconsistencies observed in *in vitro* and *in vivo* studies by chalking out alternate ways like 3D cancer models. Thus, from this extensive literature review our team aims to develop existing nanomedicines with enhanced drug delivery characteristics along with the design of a clinical trial model based on a rodent tumor model that is of close resemblance to human physiology.

## Abbreviations

NPs: nanoparticles
EPR: enhanced permeation rate
PLA: polylactic acid
DDS: drug delivery system
NC: nanocarrier
LPN: lipid nanoparticles
LET: lauryl ester of tyrosine
MMt: montmorillonite
Au NPs: gold nanoparticles
HG NPs: hollow gold nanoparticles
ARPI: acetylated rapeseed protein isolate
CNPs: chitosan nanoparticles
HPMA: *N*-(2-hydroxypropyl) methacrylamide
MNPs: ferric oxide nanoparticles
GaBS NPs: glycyrrhizic acid-biotin-starch NPs
FCA: fluorescence correlation spectroscopy
CBSA: cationic bovine serum albumin
ESC8: estrogenic derivative
(IC)_10_: indocyanine
ABC transporters: adenosine triphosphate binding cassette transporters
MDR: multidrug resistance
ECM: extracellular matrix
HRNM: hierarchical responsive nanomedicine
NIR: near infrared
DOX: doxorubicin
ICG: indocyanine green
QDs: quantum dots
NHL: non-Hodgkin lymphoma
TEM: transmission electron microscopy
SEM: scanning electron microscopy
AFM: atomic fluorescence microscopy
FRET: Förster energy resonance transfer
PTT: photothermal therapy
BSA: bovine serum albumin
LDH: amine modified layered double dihydroxide
Au NCs/Cy5.5-BSA-NPs: gold nanocluster loaded albumin nanoparticles
NM: lymph node metastasis
MiRNA: MicroRNAs
PAMAM: fourth generation poly(amidoamine) with thiol at the terminal end
PEG: polyethylene glycol
PLD: PEGylated liposomal doxorubicin
ELR: electro spun release
ED: embryonic lethal/mesodermal defects
PTX: paclitaxel
scFv: single chain antibody fragments
Fabs: antigen binding fragments
Hsp: heat shock protein
BBB: blood–brain barrier
LCT: lower critical solution temperature
UCT: upper critical solution temperature
PMIL: photoactive multi-inhibitor nano liposomes
VEGEF: vascular endothelial growth factor
ROS: reactive oxygen species
HA: hyaluronic acid
